# Simultaneous detection and quantification of DNA and protein biomarkers in spectrum of cardiovascular diseases in a microfluidic microbead chip

**DOI:** 10.1007/s00216-019-02199-x

**Published:** 2019-11-23

**Authors:** Franziska Dinter, Michał Burdukiewicz, Peter Schierack, Werner Lehmann, Jörg Nestler, Gregory Dame, Stefan Rödiger

**Affiliations:** 1 Brandenburg University of Technology Cottbus—Senftenberg, Universitätsplatz 1, 01968 Senftenberg, Germany; 2 Faculty of Mathematics and Informations Science, Warsaw University of Technology, plac Politechniki 1, 00-661 Warsaw, Poland; 3 attomol GmbH, Schulweg 6, 03205 Bronkow, Germany; 4 BiFlow Systems GmbH, Technologie-Campus 1, 09126 Chemnitz, Germany; 5 Institute of Microbiology and Virology—Brandenburg Medical School Theodor Fontane, Universitätsplatz 1, 01968 Senftenberg, Germany; 6 Faculty of Health Sciences, joint Faculty of the Brandenburg University of Technology Cottbus – Senftenberg, the Brandenburg Medical School Theodor Fontane and the University of Potsdam, Berlin, Germany

**Keywords:** Microfluidic, Multiplex, Microbeads, Cardiovascular disease, Biomarker, Autoantibodies

## Abstract

The rapid and simultaneous detection of DNA and protein biomarkers is necessary to detect the outbreak of a disease or to monitor a disease. For example, cardiovascular diseases are a major cause of adult mortality worldwide. We have developed a rapidly adaptable platform to assess biomarkers using a microfluidic technology. Our model mimics autoantibodies against three proteins, C-reactive protein (CRP), brain natriuretic peptide (BNP), and low-density lipoprotein (LDL). Cell-free mitochondrial DNA (cfmDNA) and DNA controls are detected via fluorescence probes. The biomarkers are covalently bound on the surface of size- (11–15 μm) and dual-color encoded microbeads and immobilized as planar layer in a microfluidic chip flow cell. Binding events of target molecules were analyzed by fluorescence measurements with a fully automatized fluorescence microscope (end-point and real-time) developed in house. The model system was optimized for buffers and immobilization strategies of the microbeads to enable the simultaneous detection of protein and DNA biomarkers. All prime target molecules (anti-CRP, anti-BNP, anti-LDL, cfmDNA) and the controls were successfully detected both in independent reactions and simultaneously. In addition, the biomarkers could also be detected in spiked human serum in a similar way as in the optimized buffer system. The detection limit specified by the manufacturer is reduced by at least a factor of five for each biomarker as a result of the antibody detection and kinetic experiments indicate that nearly 50 % of the fluorescence intensity is achieved within 7 min. For rapid data inspection, we have developed the open source software *digilogger*, which can be applied for data evaluation and visualization.

**Electronic supplementary material:**

The online version of this article (10.1007/s00216-019-02199-x) contains supplementary material, which is available to authorized users.

**Figure:**
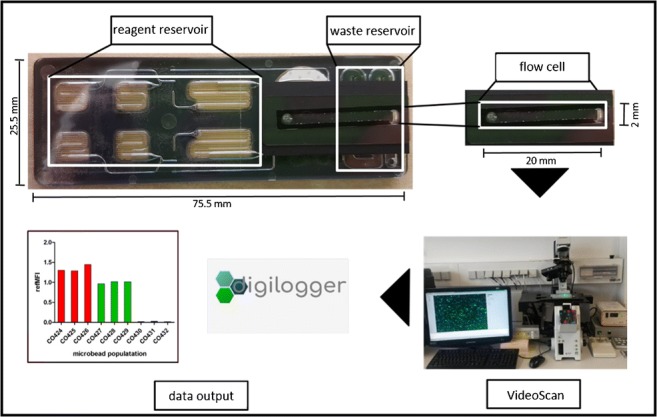
Graphical abstract

## Introduction

In order to relieve the burden on the health care system and thus avoid late diagnoses and deaths of patients, rapid identification and treatment of risk factors is necessary [1]. Biomarkers are indicator tools to assess physiological, pathogenic, or pharmacological processes.

They are used to monitor or detect diseases [2]. Prerequisites for biomarkers are high specificity, easy accessibility for high diagnostic sensitivity, high stability, and long plasma half-life [3]. For example, cardiovascular diseases (CVD) are one of the major reasons of mortality and morbidity worldwide caused by genetic predisposition, lifestyle, high misdiagnosis, and lack of clearly defined risk assessment criteria [3–5]. Immunoassays belong to the most import type of analytical methods in clinical environments and a large number of biomarkers have been investigated in CVD over the last 10 years [6, 7]. This includes C-reactive protein (CRP), brain natriuretic peptide (BNP), and low-density lipoprotein (LDL). However, there are new DNA-based biomarkers such as cell-free mitochondrial DNA (cfmDNA) that indicate systemic inflammation [8]. High concentrations of cfmDNA lead to cytokine production in monocytes and thus to an induced inflammatory reaction. This reaction may be involved in age-related CVD diseases such as heart failure, atherosclerosis, and ischemic heart disease [8, 9].

In recent years, there has been an ongoing discussion about the influence of autoantibodies on the pathology of inflammatory cardiovascular disease [11]. Several studies suggest that autoantibodies play a role in the development of cardiovascular disease, regardless of whether the person is affected by autoimmune disease or not [12–14]. In systemic lupus erythematosus (SLE), a multiorganic inflammatory disorder, CRP levels can be very low despite inflammatory activity. However, autoantibodies against CRP occur, which may be a link to acute coronary syndrome and thus a link between SLE and an increased risk of CVD [15–17]. Another autoantibody involved in SLE is anti-oxidized LDL (oxLDL). Increased detection of anti-oxLDL indicates atherosclerosis and may also serve as a marker for cardiovascular disease [18–20].

To detect several biomarkers simultaneously for a quick diagnostic statement, a combination of microfluidic and microbead technology is proposed. In comparison to conventional analytical methods, offer these technologies advantages in terms of multiplexing, time reduction, the use of smaller reaction quantities, and automation [7, 21]. The application of microbeads is suitable for the observation of the health condition of patients by a fast, multiparametric, economical, and sensitive sample analysis. The integration of microbeads in microfluidic devices brings further advantages such as reaction optimization. This can be achieved by volume reduction, meaning the use of small reaction volumes in the pico- and microliter range. Furthermore, high surface-to-volume ratios increase sensitivity; low local temperature fluctuations enable higher assay reproducibility and reduced incubation time [21]. In addition, microfluidic integration poses to potential for point-of-care testing by running an assay completely automatically in self-contained cartridges without human interference.

We demonstrate the rapid integration of putative cardiovascular biomarkers, mimicking autoantibodies against CRP, BNP, and LDL, as well as the integration of cfmDNA detection, into a microfluidic system through patient-oriented data collection, measurements, and data analysis. We show how to combine microbeads and a microfluidic technology to study the molecular interaction between antibody and antigen as well as between DNA and DNA (Table 1).

**Table 1:** Biomolecules application in microbead-based microfluidic chip. CRP, BNP, and LDL served as model targets to mimic autoantibody reactions

Biomolecule	Term and sequences	Detection molecule	Application
CRP	C-reactive protein	Monoclonal antibody (IgG) against CRP, Cy5 conjugated	Protein-based cardiovascular biomarker immunofluorescence dilution 1:50–1:200
BNP	Brain natriuretic peptide	Monoclonal antibody (IgG) against BNP, Cy5 conjugated	Protein-based cardiovascular biomarker immunofluorescence dilution 1:50–1:200
LDL	Low-density lipoprotein	Polyclonal antibody (IgG) against LDL, Cy5 conjugated	Protein-based cardiovascular biomarker immunofluorescence dilution 1:50–1:200
cfmDNA	Cell-free mitochondrial DNA 5′-TGG GAG TGG GAG GGG AAA ATA ATG TGT TAG TTG GGG GGT GAC TGT TAA AAG TGC ATA CCG CCA AAA GAT AAA ATT TGA AAT CTG GTT AGG CTG GTG TTA GGG TTC TTT GTT TTT GGG GTT TGG CAG AGA TGT GTT TAA GTG CTG TGG CCA-3′	Capture probe 5′-(T)_20_ATCTCTGCCAAACCCC-3′3′biotinylated detection probe 5′-TTGGCGGTATGCACTT-3′, 5′Cy5 conjugated	DNA-based cardiovascular biomarker
FISH	Capture probe 5′-(T)_20_ACWCCTACGGGWGGCWGC-3′, 3′biotinylated	EUB338 5′-GCWGCCWCCCGTAGGWGT-3′, 5′Cy5 conjugated [10]	Unrelated internal control from fluorescence *in situ* Hybridization experiments
HPV72	Human papilloma virus 72 5′-CATCTGTTGGTTTAATGAGCTT-3′, 3′Cy5 conjugated, 5′biotinylated	Detection probe 5′-AAGCTCATTAAACCAACAGATG-3′, 3′biotinylated	Unrelated internal control
SAv	Streptavidin		Negative control

## Experimental

### Microfluidic chip

**Fig. 1:**
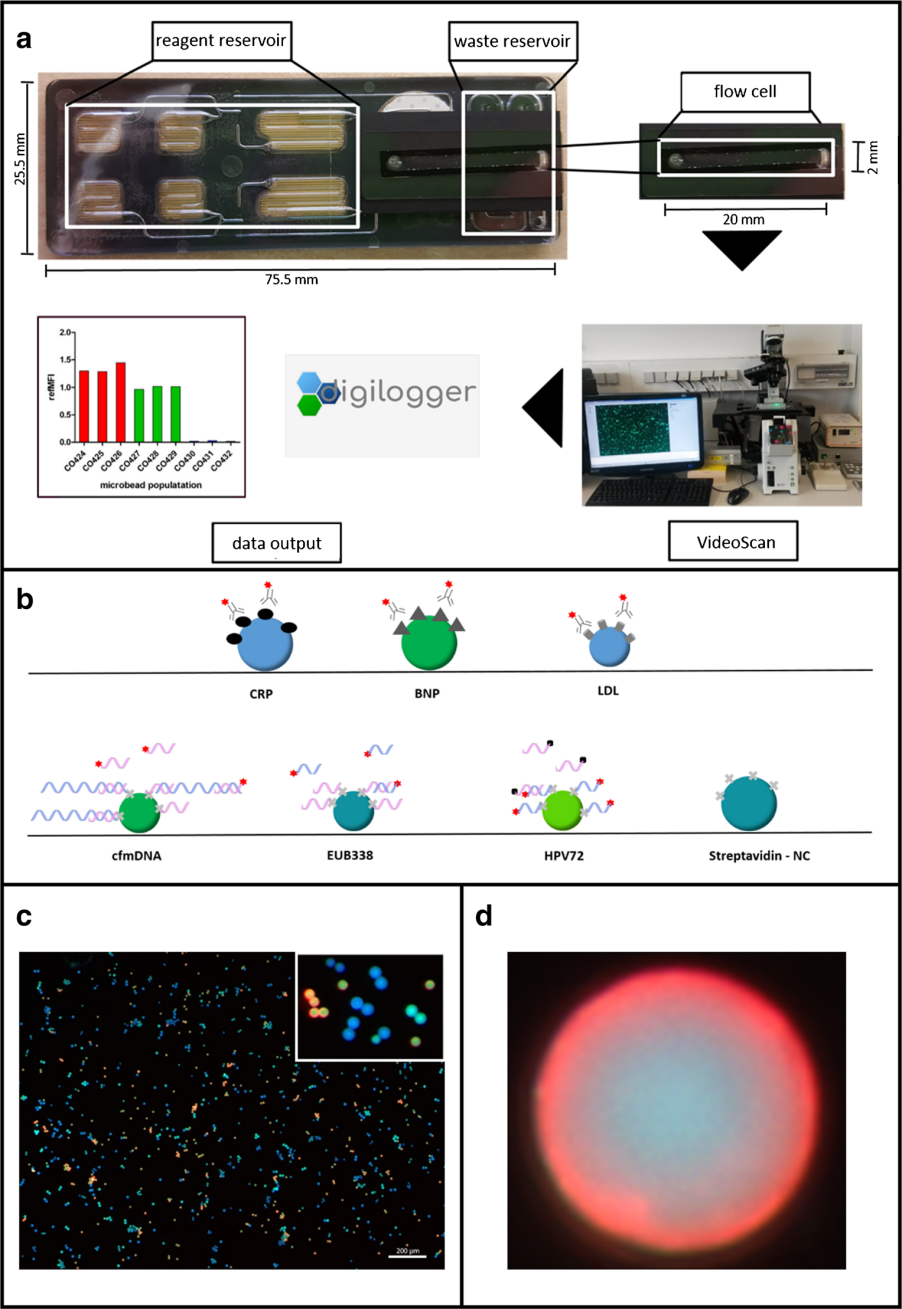
Microfluidic microbead chip system for the analysis of cardiovascular diseases**. a** In microfluidic chips (flex.flow, BiFlow Systems GmbH), microbeads modified by us were immobilized in a random arrangement on a carrier substrate in the flow cell. Reagent reservoirs in microfluidic chip are filled with assay components (e.g., buffer, sample solution). During the assay, the components are pumped via Bi.Flow Systems′ pump control software through microfluidic channels. After incubation and washing steps, the microbeads were measured in the flow cell using VideoScan technology (Bioimage informatics). The surface fluorescence intensity results from a molecular interaction of the target molecules with the detector molecules and is expressed as referenced mean fluorescence intensity (refMFI). The data are then evaluated with the digilogger software (video in Electronic Supplementary Material (ESM)). **b** Each microbead population encodes one target molecule CRP, BNP, LDL and cfmDNA. Those target molecules are detected by fluorescence labelled antibodies or DNA probe. **c** Furthermore, the microbeads are coded by fluorescence and size, shown here in false colors. **d** A detection of the target molecule becomes visible at the microbead through a red corona

We used a commercially available microfluidic chip consisting of six reagent reservoirs with volumes of 25 and 50 μL, a bubble trap, and a waste reservoir (flexflow slides, BiFlow Systems GmbH, Germany). The reservoirs can be emptied by integrated electrochemical micropumps. The technical setup and operation principle of the microfluidic cartridges as well as of the integrated micropumps is described elsewhere [22–24]. One chip can be used for one patient. The reagent reservoirs were filled with assay components (buffers or sample solution). Reagent reservoirs and flow cell were sealed with pressure-sensitive adhesive cover tape. The integrated micropumps and thus the volume flows in the cavities were controlled via the pump control software (Fig. 1). A video on the handling of the microfluidic chip can be found in the ESM.

### Microbeads

We used seven carboxylated poly(methylmethacrylate) (PMMA) microbead populations (PolyAn GmbH, Germany). The microbeads are coded by two fluorescence dyes (Rhodamin 6G and Coumarin 334) and by size (Table 2) [25, 26].

**Table 2:** Assignment of biomarkers to microbeads

Microbead population	Diameter	Loading
MB1	11.2 μm	CRP
MB2	11.1 μm	BNP
MB3	11.0 μm	LDL
MB4	14.4 μm	cfmDNA
MB5	15.0 μm	Streptavidin
MB6	15.4 μm	EUB338 (FISH)
MB7	12.3 μm	HPV72

### Covalent coupling of biomarkers on microbeads

Independently, proteins CRP (antibodies-online GmbH, Germany), BNP (Bachem, Switzerland), LDL (Biozol, Germany), and streptavidin (internally produced according to [27]) were covalently bound to microbeads (Table 2) using a crosslinker 1-ethyl-3- (3- dimethylaminopropyl) carbodiimid hydrochloride (EDC, Karl Roth, Germany). Streptavidin was used for the immobilization of biotinylated DNA probes. Per population 10^5^ microbeads were washed first with 200 μL 2-(N-morpholino)ethanosulfonic acid 0.1 M (MES, pH 4.6, Karl Roth, Germany). Afterwards, carboxyl groups were activated by shaking for 20 min at 28 °C and 1200 rpm with EDC solution (25 mg/mL, dissolved in MES). Followed by washing step with 0.05 × phosphate-buffered saline (PBS) and incubated by shaking (3 h at 28 °C 1200 rpm) with corresponding protein (300 μg/μL, dissolved in 0.05 × PBS). Microbeads were washed three times with 200 μL tris-buffered saline containing 0.1 % Tween (TBS-T).

### Coupling of capture probes on microbeads

The biotinylated capture probes for cfmDNA (cfmDNA CP, biomers.net GmbH, Germany) EUB338 (unrelated internal control, biomers.net GmbH, Germany) and HPV72 (unrelated internal control, biomers.net GmbH, Germany) were independently coupled to streptavidin coated microbeads (MB4, MB6, and MB7) at 50 nM concentration (1 h at 28 °C and 1200 rpm). Afterwards the microbeads were washed three times with 200 μL TBS-T to remove unbound capture probes (see ESM, Supp Sec 1, Fig. S1).

### Assay preparation

All coated microbead populations (MB1–MB7) were pooled in equal parts. A mixture of approximately 2000 microbeads was immobilized with immobilization solution (Medipan GmbH, Germany) on flow cell surface on microfluidic chip and dried over night at RT. The multiwell plate does not have to be prepared.

### Protein assay—mimicking autoantibody testing

For protein detection (mimicking autoantibodies) in microfluidic chip, reagent reservoirs were filled with TBS-T buffer and 20 ng/μL of corresponding antibody (anti-CRP monoclonal antibody conjugated with Cy5 (Bioss antibodies, USA), anti-BNP monoclonal antibody conjugated with Cy5 (Bioss antibodies, USA)). First, TBS-T was pumped through the flow cell to create a wet environment (see ESM, Supp Sec 2, Table S1). This was followed by a basis microbead measurement using VideoScan technology [26]. Then, antibody solution was pumped through flow cell and was incubated for 1 h at RT in the dark. To remove the excess of antibodies in flow cell, the microbeads were washed once with TBS-T, followed by final fluorescence measurement in VideoScan technology. For protein detection in the multiwell plate, the assay was performed in the same order as in the chip. However, the microbead mix was incubated with the corresponding antibody for 1 h at RT and 1200 rpm in the thermomixer and then washed three times with TBS-T. One washing step consisted of a centrifugation step (1 min at 10,000 rpm), the aspiration of the supernatant and the subsequent addition of 200 μL TBS-T. The microbeads were then transferred to a multiwell plate and measured using VideoScan technology. For dilution series, the assay was performed with different concentrations (0.2 ng/μL to 100 ng/μL) of the corresponding antibody.

In addition, the biomarkers were spiked into human serum (offered from donor) in a concentration range from 0.2 to 50 ng/μL and detected with the aid of microbeads in chip assay as well as in multiwell plate.

### DNA assay for cfmDNA testing

Reagent reservoirs of microfluidic chip were filled with TBS-T, cfmDNA (10 ng/μL, Biotez Berlin Buch GmbH, Germany) and detection probe for cfmDNA (cfmDNA DP, 50 nM, Cy5 conjugated, Biomers.net GmbH, Germany). As described in subsection before (“Protein assay—mimicking autoantibody testing”), the method was equally performed until step of basis measurement. cfmDNA solution was pumped through flow cell and incubated for 1 h at RT in the dark. To remove the excess of cfmDNA in flow cell, the microbeads were washed once with TBS-T. Afterwards, the cfmDNA DP solution was pumped through flow cell and incubated for 1 h at RT in the dark. After washing with TBS-T, the fluorescence signal was measured in VideoScan technology. In the multiwell plate, the assay was performed in the same order as in the chip. The incubation steps with the cfmDNA to be detected and cfmDNA DP were carried out for 1 h at RT and 1200 rpm in the thermomixer, interrupted by washing with TBS-T three times each.

The microbead suspension was then transferred to a multiwell plate and measured using VideoScan technology. For dilution series, the assay was performed with different concentrations (0.5 to 100 ng/μL) of the cfmDNA.

In addition, the cfmDNA was spiked into human serum (offered from donor) in a concentration range from 0.05 to 50 ng/μL and detected with the aid of microbeads in chip assay as well as in multiwell plate.

### Kinetics

Microfluidic chips for kinetic experiments were prepared as described before. The fluorescence signal was measured every 3 min during 1 h incubation of antibody solution in protein assay and cfmDNA DP incubation in DNA assay.

### Data analysis

Images were acquired using the VideoScan platform and the image data analyzed using the FastFluoScan software (see [26] section 2.1–2.2). The proper linear range of fluorescence intensity was determined by an adaptive algorithm. Details can be found in the ESM (Supp Sec 5.) All raw data (shown in ESM in files Raw_data.xlsx and Serum_Data.xlsx) were analyzed with the R statistical [28] computing environment. Non-linear kinetic curve data were fitted using the *drc* package [29] and plotted with the 95 % confidence interval (see ESM, Supp Sec 6–9). The biomolecular interaction is reported as refMFI (referenced fluorescence intensity, see [26] for details). Precision medicine yields a large amounts of data that surpasses human ability to understand it [30]. Therefore, there is a growing need of a dedicated software streamlining getting the gist of out the data. For this project, we developed the graphical user interface *digilogger* as R package (https://github.com/michbur/digilogger) that eases the visual exploration of the data (see ESM, Supp Sec 10).

## Results and discussion

Conventional detection methods of biomarkers are single detection by immunoassay (membrane or chip based) or qPCR [31, 32]. Growing attention is paid to the role of autoantibodies in CVD [11, 33]. We have developed a model system, to simultaneously measure and quantify protein and DNA biomarkers in a microfluidic microbead chip. Experiments have been carried out to demonstrate the functionality of our model, including the optimization of the system through a suitable buffer system, an applicable immobilization method, and the analysis of biomarkers in human serum.

### Optimization of microbead immobilization and assay environment

Optimal assay conditions in a microfluidic chip are based on an optimized microbead immobilization. Three options (method A, immobilization by drying; method B, poly-L-lysine (PLL); method C, immobilization solution (Medipan GmbH)) were tested. The end point was the lowest microbead loss after all washing steps. The immobilization of the microbeads by method A showed the highest loss of microbeads with 37 %. Method B had the lowest microbead loss of 6 %. The immobilization of microbeads with PLL was described as the most effective method in [26]. However, unspecific binding of oligonucleotides to the coated surface was observed. Thus, method B was discarded. Microbead immobilization with method C resulted in ~ 25 % microbead loss. No unspecific binding was observed. Thus, method C was further used to immobilize the microbeads on chip surface.

Next, we optimized the buffer system (see ESM, Supp Sec 3, Fig. S2). Four different buffer systems (PBS, PBS-T, TBS, and TBS-T) were used. All biomarkers were tested in single measurements in each buffer. Both PBS-T and TBS-T were found to be suitable for further use. Using PBS and TBS without the surfactant and spreading agent Tween 20 (Polysorbate 20), only low signals were obtained or microbeads aggregated in the flow cell. Finally, TBS-T was selected as buffer system for further experiments, since the use of phosphate-buffered systems can lead to unspecific binding [34].

### Proof of concept for the detection of biomarkers in microfluidic microbead chip system

**Fig. 2:**
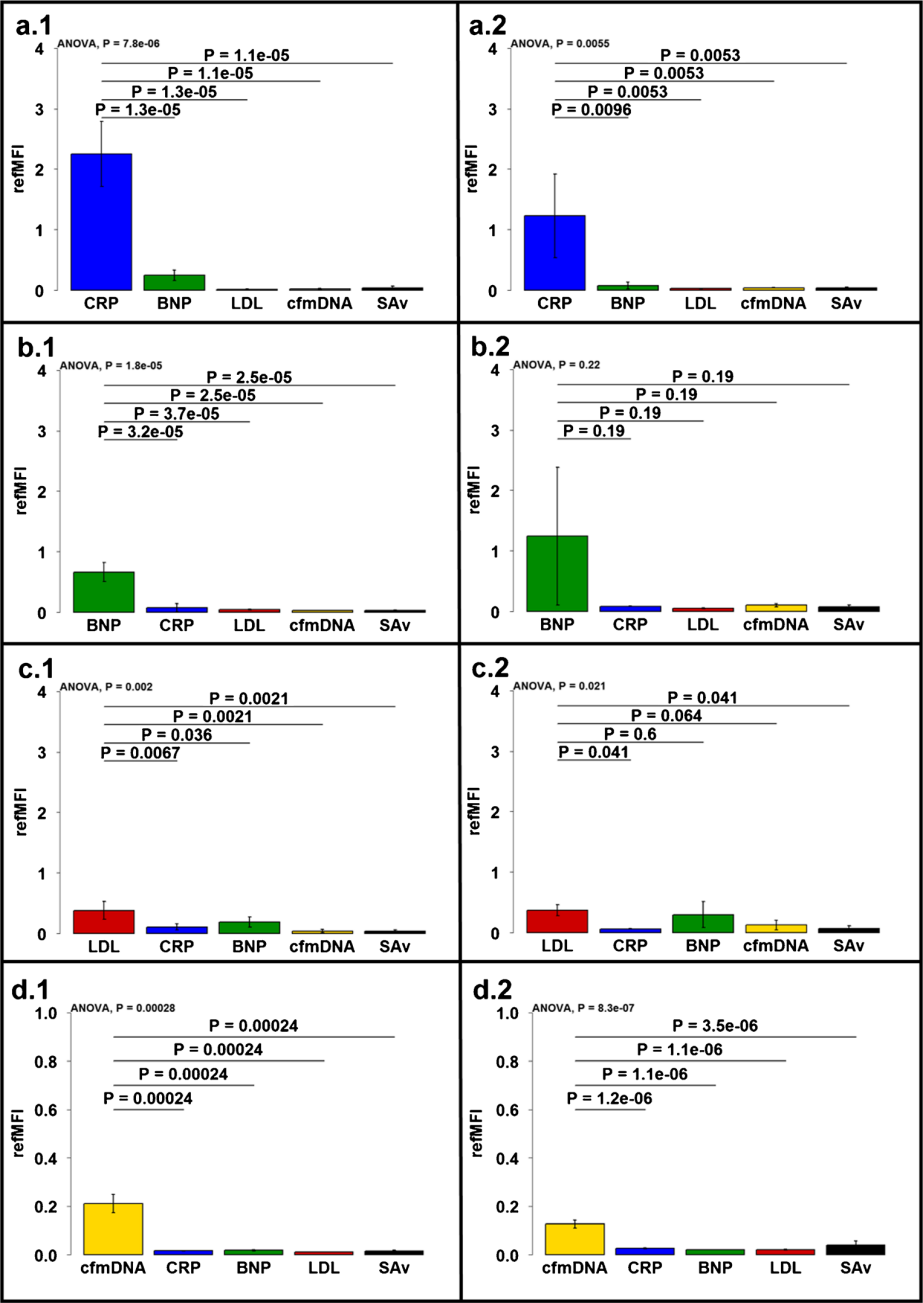
Single measurements of cardiovascular biomarkers in chip system and multiwell plate. The specificity of the individual biomarkers CRP (**a**), BNP (**b**), LDL (**c**), and cfmDNA (**d**) was determined in single measurements in the chip system (a.1–d.1) as well as in a multiwell plate (a.2–d.2). A microbead mix of all coupled microbead populations was used and incubated with the respective biomarker. All results are shown with significances in the figure

**Fig. 3:**
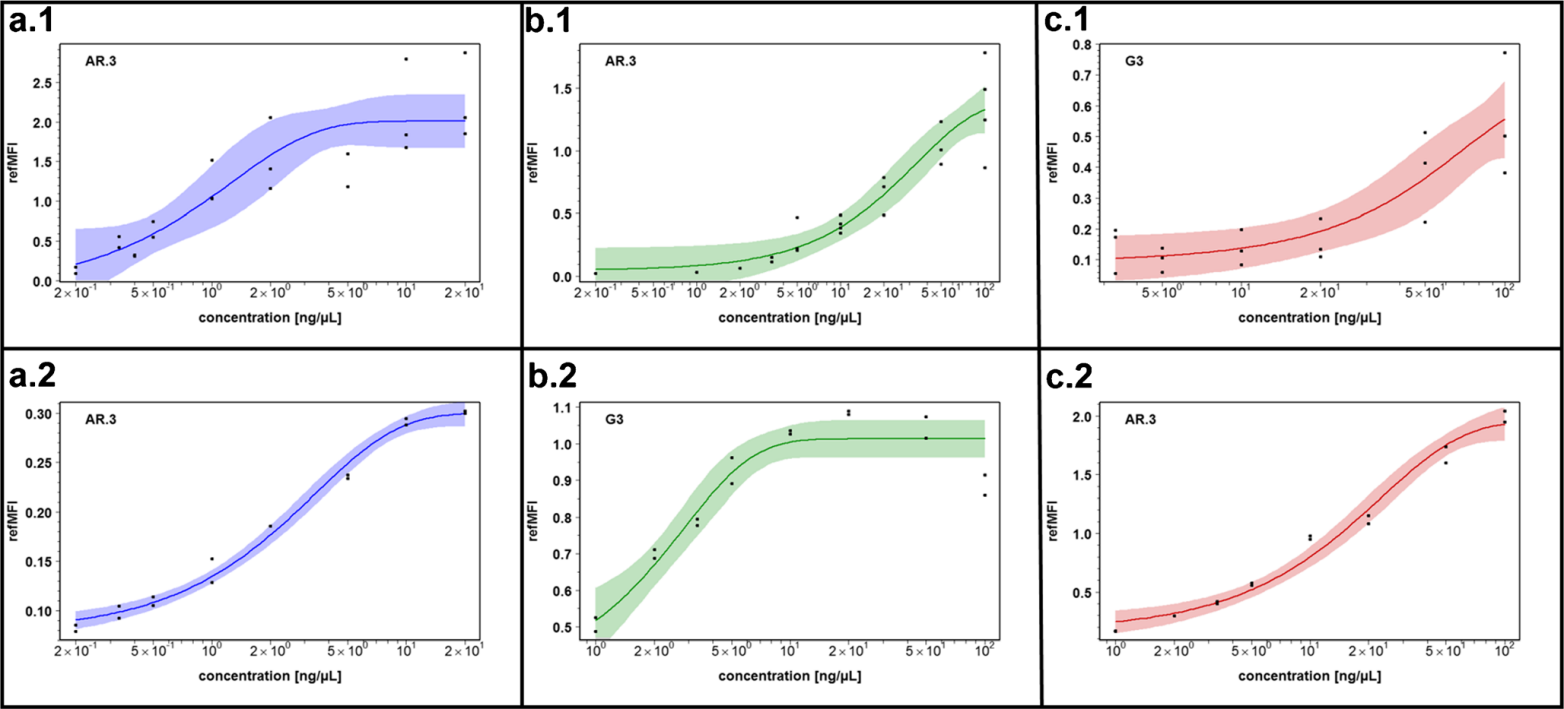
Serial dilution of CRP, BNP, and LDL in chip and multiwell plate. The sensitivity of all detection molecules was determined by dilution series in the chip (a.1–c.1) and in the multiwell plate (a.2–c.2). Different concentrations of antibodies from 0.2 to 100 ng/μL were used. The representation of the curves (95 % confidence interval) was made using statistical models gompertz (G3) and asymptotic regression (AR.3)

Autoantibodies appear to play a role in CVD [11–14]. During the development of a model system for the simultaneous detection of protein and DNA biomarkers in a microbead-based microfluidic chip, the proteins CRP, BNP, and LDL were coupled to corresponding microbeads (antigen) and detected by a specific fluorescence labelled antibody (~autoantibody). This system was used to mimick autoantibodies. For the detection of the DNA-based biomarker, a capture probe was coupled to a microbead population, which captures the cfmDNA and is then detected by a second fluorescence labelled probe. To determine the specificity (Fig. 2) and sensitivity (Fig. 3) of individual detection molecules, all biomarkers (anti-CRP, anti-BNP, anti-LDL, and cfmDNA) were detected in single reactions both in the chip (Fig. 2a.1–d.1) and in the multiwell plate (Fig. 2a–d). For this purpose, a mix of coupled microbead populations (MB1–MB5, in equal quantities) was applied in corresponding assays. Anti-CRP, anti-BNP, anti-LDL and cfmDNA are specifically detectable in all single reactions in the chip, but both the anti-CRP antibody and the anti-LDL antibody recognize BNP unspecifically. All values obtained with the chip system are significant, whereas the values from BNP detection obtained from multiwell plate show no significant differences in fluorescence signal. The detection of cardiovascular biomarkers in the multiwell plate is specific except for the detection of LDL, where BNP is detected by the anti-LDL antibody and does not provide a significant signal difference to detected LDL. In comparison to the measurement in multiwell plate, higher ligand values are achieved using a chip system by a factor of 1.9 for CRP and by a factor of 1.7 for cfmDNA. The ligand values for the detection of LDL barely differ in the comparison of both methods, whereas BNP reaches ligand value in the multiwell plate that is 1.7 times higher, but with a high standard deviation. Looking at the signal-to-noise ratios ($\mathrm{SNR}=10{\log}_{10}\left(\frac{\mathrm{signal}}{\mathrm{noise}}\right)$ [35]), the chip system shows a better result as all signals have a higher dB value. CRP (chip, 16.96 dB; plate, 15.67 dB), BNP (chip, 13.84 dB; plate, 12.04 dB), LDL (chip, 13.19 dB; plate, 7.93 dB), and for cfmDNA (chip, 11.46 dB; plate, 5.85 dB). Here, an advantage is shown in the lower background signal of the chip in contrast to the multiwell plate. Using dilution series, the sensitivity of each individual detection molecule was tested both in the chip and in the multiwell plate (Fig. 3). The detection limit for the anti-CRP antibody is 0.2 ng/μL (refMFI 0.137) in the chip and 0.5 ng/μL (refMFI 0.1) in the multiwell plate. This corresponds to a detection limit lower by a factor of 25 (chip) and 10 (plate) than the manufacturer’s values (Biozol, 5 ng/μL). The detection limits for BNP and LDL in the chip are 3.33 ng/μL each (BNP–refMFI 0.134, LDL–refMFI 0.14) and 1 ng/μL in the plate (BNP–refMFI 0.5, LDL–refMFI 0.163), which is 1.5 times (chip) and 5 times lower than specified by the manufacturer (Biozol, 5 ng/μL). The detection limit for the DNA-based biomarker cfmDNA is 0.5 ng/μL (refMFI 0.09) in the chip and 0.1 ng/μL (refMFI 0.09) in the multiwell plate. Dilution series of cfmDNA was performed as an example only and is therefore shown in the ESM (Supp Sec 4, Fig. S3). In comparison to the detection limits stated by the manufacturer, a microbead-based system can be used with higher antibody dilutions both in the chip and in the multiwell plate. Thus, we consider this as a promising tool for autoantibody testing against CRP, BNP, and LDL. The biomarkers were detected simultaneously in one dilution series (see ESM, Supp Sec 4, Fig. S4). This shows that the values in the chip during simultaneous measurement for CRP (simultaneous, refMFI 1.32; separately, refMFI 0.65) and BNP (simultaneous, refMFI 0.36; separately, refMFI 0.13) are approximately twice as high as in the separate measurement, whereas the values for LDL are almost identical in both the separated measurement and the simultaneous measurement (simultaneous, refMFI 0.14; separately, refMFI 0.14). The values obtained in the multiwell plate show no differences in the comparison of the two methods, CRP (simultaneous, 0.19; separately, refMFI 0.1), BNP (simultaneous, refMFI 0.82; separately, refMFI 0.78), and LDL (simultaneous, refMFI 0.18; separately, refMFI 0.16). To test the assay functionality under non-optimal conditions, human serum was spiked with antibodies against CRP, BNP, LDL, and cfmDNA. All biomarkers could be detected on the respective microbeads without non-specific binding. Again, differences in fluorescence signal were observed between the chip system and the multiwell plate. The previously shown results under optimal buffer conditions could be confirmed using human serum (see ESM, Supp Sec 4, Fig. S5).

### Fluorescence intensity determination as a function of time

**Fig. 4:**
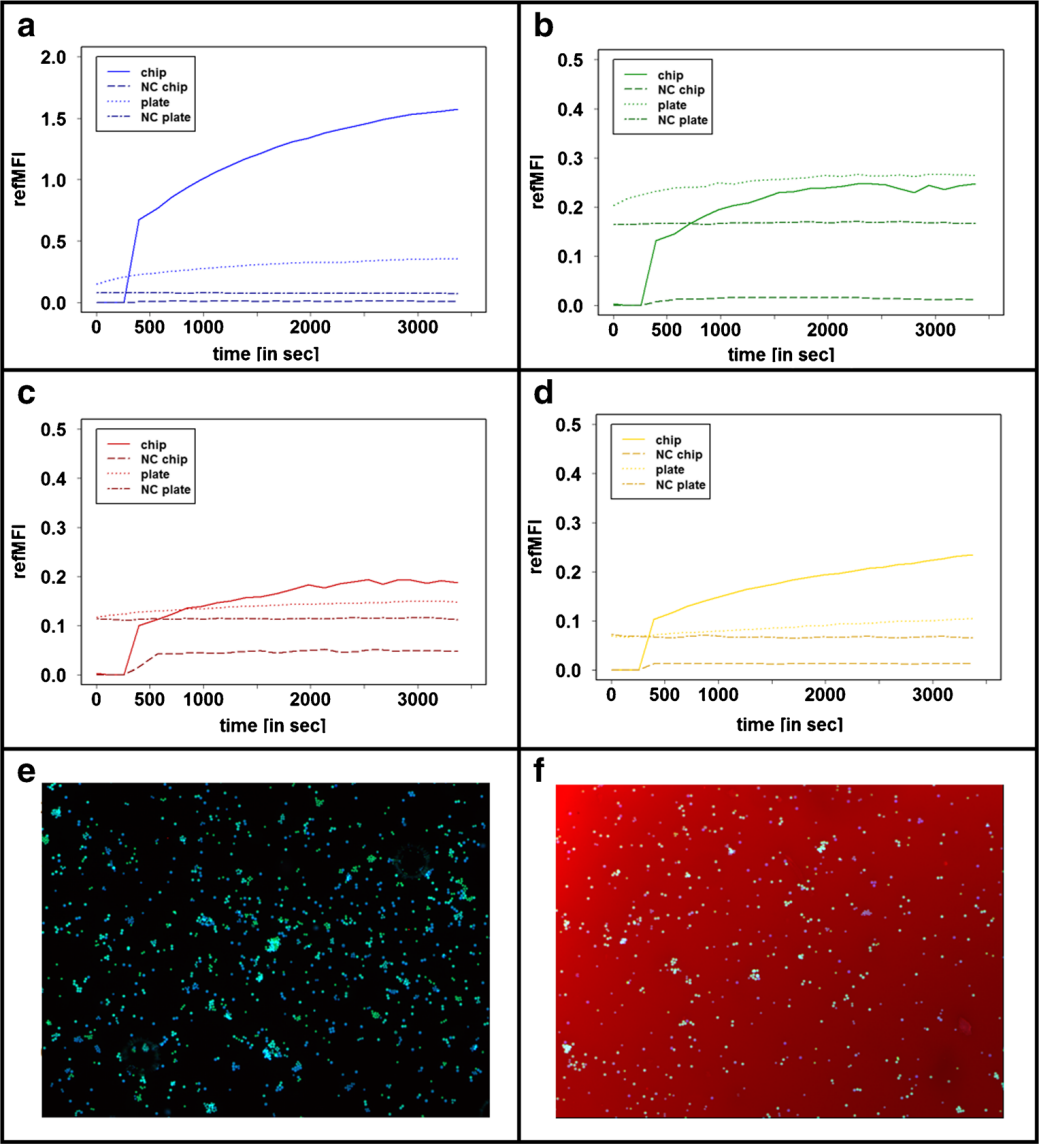
Fluorescence intensity determination as a function of time. Time-dependent detection of the biomarkers anti-CRP (**a**), anti-BNP (**b**), anti-LDL (**c**), and cfmDNA (**d**) as comparison in the microfluidic chip and in the multiwell plate. The range in the chip measurement from 0 to 400 s indicates the injection of the assay components into the flow cell. Images taken during the measurement show the flow cell of the **e** chip as well as **f** the multiwell plate. The red coloration in figure **f** represents the high background signal in the multiwell plate

Kinetics experiments are used to document the temporal course of the signal rise of an assay (Fig. 4). The fluorescence intensity of the microbeads was measured over a period of 1 h. For the chip, an initial measurement was performed before the detection molecule was pumped through the capillary of the chip. After a pumping time of 400 s, the fluorescence intensity was measured repeatedly. For all biomarkers, approximately half of the maximum fluorescence intensity was reached. A time-dependent signal increase was visible for all biomarkers. The detection of CRP after 1 h incubation approached a maximum fluorescence intensity of refMFI 1.57, BNP of refMFI 0.24, LDL of refMFI 1.96, and cfmDNA of refMFI 0.23. The fluorescence intensity of the microbeads in the multiwell plate was incubated from the zero value for 1 h and also measured in 3 min intervals. Compared to measurements in the chip, no initial value of zero is reached here, which indicates a high background signal. The increase in fluorescence intensity as a function of time is also not visible. Maximum fluorescence signals are calculated for CRP refMFI 0.37, BNP refMFI 0.26, LDL refMFI 0.14, and for cfmDNA refMFI 0.1.

### Simultaneous detection of protein and DNA-based biomarker in a microfluidic microbead chip

**Fig. 5:**
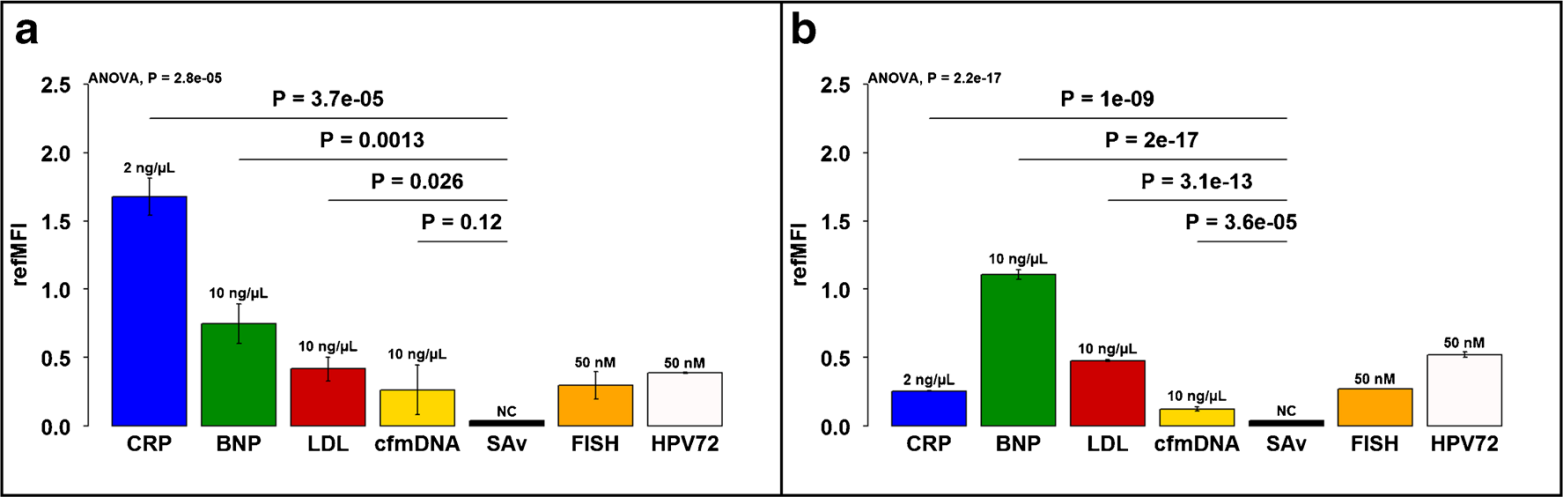
Simultaneous detection of protein and DNA-based cardiovascular biomarker. The simultaneous detection of the biomarkers CRP, BNP, LDL, and cfmDNA in one chip (**a**) and in the multiwell plate (**b**) was complemented by the DNA-based detection molecules EUB338 (FISH) and HPV72

To ensure a fast and precise diagnosis, it is necessary to be able to detect several biomarkers simultaneously in one sample. Furthermore, it is advantageous to combine the detection of two different molecule classes (e.g., protein, DNA) to expand the variety of biomarkers and to save sample material [36]. All biomarkers (anti-CRP, anti-BNP, anti-LDL, and cfmDNA) and the additional DNA control molecules HPV72 and EUB338 (FISH) were detected simultaneously in the chip and in a multiwell plate (Fig. 5). The simultaneous detection of the biomarkers confirms the tendency of the results of the individual detections (Fig. 2) both from the chip and from the multiwell plate. Anti-CRP antibodies are used to detect the protein CRP with a fluorescence signal 6.5 times higher in the chip than in the multiwell plate. Anti-BNP detects the protein in the multiwell plate with a fluorescence signal 1.6 times higher than in the chip. The detection of LDL achieves almost identical results using both methods. cfmDNA is detected via the detection probe in the chip with a fluorescence signal twice as high as in the multiwell plate. The results for BNP, LDL, and cfmDNA are almost identical in direct comparison to the individual biomarker measurements. In contrast, the fluorescence signal for CRP is reduced by half for both methods. It can be said that the detection of individual biomolecules is not affected by the simultaneous detection of six different molecules within a cavity, also seen in S4. Could an improved binding behavior due to the prevailing competition of the individual molecules as well as the high binding affinity of antibodies to the antigen and DNA to the DNA be possible reasons for a higher fluorescence signal?

### Comparison of microfluidic microbead chip and conventional multiwell plate

In the comparison of the detection of CRP, BNP, LDL, and cfmDNA in the microbead microfluidic chip and the multiwell plate, the use of the chip shows more advantages compared to the multiwell plate. Lower volumes are used in the chip, so it is possible to save patient material. The chip is fully automatized (e.g., reproducible volume flows) and removes human bias. Furthermore, we observed lower background signals in the chip despite fewer washing steps than in the multiwell plate. The lower background is due to the fact the fluorescence sum signal is lower in the chip (300 μm chamber height) then in the multiwell plate (2000 μm liquid column). Kinetics showed that approximately 50 % of the maximum fluorescence intensity is achieved after an incubation time of 7 min. Signals in the multiwell plate needed 1 h. In everyday laboratory use, the microfluidic chip has a disadvantage in terms of costs and handling factor. The chip can only be used once and needs several manual preparation steps. However, both factors can be minimized in the later high-throughput process. Since the microfluidic chips have a lower cost on a higher scale. The effort in the preparation steps can also be reduced by a dispenser and the automatic filling of the chips. A comparison between chip and multiwell plate can be found in Table 3.

**Table 3:** Comparison of microfluidic microbead chip and multiwell plate

Microfluidic microbead chip		Multiwell plate	
Advantages	Disadvantages	Advantages	Disadvantages
Low background signal Fast reaction Small reaction volumes Few washing steps High surface-to-volume ratios Increase sensitivity	Expensive (small scale) Time-consuming preparation (not automatized) Low throughput (higher hands-on-time)	Cheap High throughput Rapid preparation	High background signal Many (manual) washing steps

## Conclusion

A model system for the simultaneous detection of protein and DNA-based cardiovascular biomarkers has been successfully developed. For example, it is possible to use this system for autoantibody detection as well as for antibody titer determination. The detection of biomarkers in a microfluidic microbead chip has advantages over measurements in multiwell format, such as low background signals, small reaction volumes, and fast reaction. We have chosen a simple microfluidic structure and a low chamber containing randomly ordered microbeads. We never observed channel blockage as described by others [7]. We found the system is easy to assemble and can therefore be adapted to new tests. In order to make the system more usable, further optimizations have to be carried out. For the readout of the image data, we used the FastFluoScan software of the VideoScan system. There are alternative open source bioimage informatics software packages [37] and smartphone-based readout [38] that could be used. These include improving the reproducibility of cfmDNA detection and reducing the overall cost of a chip (including beads, biomarkers, and detection molecules). In addition, a secondary antibody or aptamers [39] for signal amplification could be integrated into the system for further experiments and the application of patient sample material with tested autoantibodies is also required. More biomarkers should be integrated into the system. Our technology theoretically allows the use of up to 18 different microbead populations.

## Electronic supplementary material

ESM 1 (PDF 2154 kb)

ESM 2 (XLSX 101 kb)

ESM 3 (XLSX 68 kb)

## References

[1] Upadhyay RK (2015). Emerging risk biomarkers in cardiovascular diseases and disorders. J Lipids.

[2] National Institutes of HealthDirector’s Initiative on Biomarkers and Surrogate Endpoints (2001). Biomarkers and surrogate endpoints: preferred definitions and conceptual framework. Clin Pharmacol Ther.

[3] McDonnell B, Hearty S, Leonard P, O’Kennedy R (2009). Cardiac biomarkers and the case for point-of-care testing. Clin Biochem.

[4] Dhingra R, Vasan RS (2017). Biomarkers in cardiovascular disease: statistical assessment and section on key novel heart failure biomarkers. Trends Cardiovasc Med.

[5] Berezin EA. Circulating cell-free mitochondrial DNA as biomarker of cardiovascular risk: new challenges of old findings. Angiol. 2015. 10.4172/2329-9495.1000161.

[6] Albert MA. Biomarkers and heart disease. J Clin Sleep Med. 2011. 10.5664/jcsm.1342. 10.5664/JCSM.1342 PMC3190417 22003335

[7] Peng S, Hong T, Liang W, Liu W, Chen C (2019). A multichannel microchip containing 16 chambers packed with antibody-functionalized beads for immunofluorescence assay. Anal Bioanal Chem.

[8] Nakayama H, Otsu K (2018). Mitochondrial DNA as an inflammatory mediator in cardiovascular diseases. Biochem J.

[9] Berezin AE (2016) DTOhI: 1e0.21C76e7/l2l1-72F-0r4e79e.100M068itochondrial DNA: a novel biomarker of cardiovascular risk? Transl Biomed 4. 10.21767/2172-0479.100068.

[10] Amann RI, Devereux R, Stahl’ DA (1990). Combination of 16S rRNA-targeted oligonucleotide probes with flow cytometry for analyzing mixed microbial populations. Appl Environ Microbiol.

[11] Meier LA, Binstadt BA. The contribution of autoantibodies to inflammatory cardiovascular pathology. Front Immunol. 2018. 10.3389/fimmu.2018.00911. 10.3389/fimmu.2018.00911 PMC5934424 29755478

[12] LIANG KIMBERLY P., MARADIT-KREMERS HILAL, CROWSON CYNTHIA S., SNYDER MELISSA R., THERNEAU TERRY M., ROGER VERONIQUE L., GABRIEL SHERINE E. (2009). Autoantibodies and the Risk of Cardiovascular Events. The Journal of Rheumatology.

[13] Müller J., Wallukat G., Schimke I. (2017). Autoantibody-Directed Therapy in Cardiovascular Diseases. The Heart in Rheumatic, Autoimmune and Inflammatory Diseases.

[14] Frostegård J (2002). Autoimmunity, oxidized LDL and cardiovascular disease. Autoimmun Rev.

[15] O’Neill SG, Isenberg DA, Rahman A (2007). Could antibodies to C-reactive protein link inflammation and cardiovascular disease in patients with systemic lupus erythematosus?. Ann Rheum Dis.

[16] Wetterö J, Nilsson L, Jonasson L, Sjöwall C (2009). Reduced serum levels of autoantibodies against monomeric C-reactive protein (CRP) in patients with acute coronary syndrome. Clin Chim Acta.

[17] O’Neill SG, Giles I, Lambrianides A, Manson J, D’Cruz D, Schrieber L, March LM, Latchman DS, Isenberg DA, Rahman A (2010). Antibodies to apolipoprotein A-I, high-density lipoprotein, and C-reactive protein are associated with disease activity in patients with systemic lupus erythematosus. Arthritis Rheum.

[18] Wilson PWF, Ben-Yehuda O, McNamara J, Massaro J, Witztum J, Reaven PD (2006). Autoantibodies to oxidized LDL and cardiovascular risk: the Framingham Offspring Study. Atherosclerosis.

[19] Vaarala O (2000). Autoantibodies to modified LDLs and other phospholipid-protein complexes as markers of cardiovascular diseases. J Intern Med.

[20] Iseme RA, McEvoy M, Kelly B, Agnew L, Walker FR, Handley T, Oldmeadow C, Attia J, Boyle M (2017). A role for autoantibodies in atherogenesis. Cardiovasc Res.

[21] Rödiger S, Liebsch C, Schmidt C, Lehmann W, Resch-Genger U, Schedler U, Schierack P (2014). Nucleic acid detection based on the use of microbeads: a review. Microchim Acta.

[22] Streit P, Nestler J, Shaporin A, Graunitz J, Otto T (2018). Design methodology and results evaluation of a heating functionality in modular lab-on-chip systems. J Micromech Microeng.

[23] Schumacher S, Nestler J, Otto T (2012). Highly-integrated lab-on-chip system for point-of-care multiparameter analysis. Lab Chip.

[24] Geidel S, Peransi Llopis S, Rodrigo M, de Diego-Castilla G, Sousa A, Nestler J, Otto T, Gessner T, Parro V (2016). Integration of an optical ring resonator biosensor into a self-contained microfluidic cartridge with active, single-shot micropumps. Micromachines.

[25] Rödiger S, Ruhland M, Schmidt C, Schröder C, Grossmann K, Böhm A, Nitschke J, Berger I, Schimke I, Schierack P (2011). Fluorescence dye adsorption assay to quantify carboxyl groups on the surface of poly(methyl methacrylate) microbeads. Anal Chem.

[26] Rödiger S, Schierack P, Böhm A, Seitz H, Schumacher S (2012). A highly versatile microscope imaging technology platform for the multiplex real-time detection of biomolecules and autoimmune antibodies. Mol. Diagn.

[27] Gallizia A, de Lalla C, Nardone E, Santambrogio P, Brandazza A, Sidoli A, Arosio P (1998). Production of a soluble and functional recombinant streptavidin in Escherichia coli. Protein Expr Purif.

[28] R Core Team (2018) R: a language and environment for statistical computing. R Found Stat Comput https://www.R-project.org/.

[29] Ritz C, Baty F, Streibig JC, Gerhard D (2015). Dose-response analysis using R. PLoS One.

[30] Prosperi M, Min JS, Bian J, Modave F. Big data hurdles in precision medicine and precision public health. BMC Med Inform Decis Mak. 2018. 10.1186/s12911-018-0719-2. 10.1186/s12911-018-0719-2 PMC6311005 30594159

[31] Adamcova M, Šimko F (2018). Multiplex biomarker approach to cardiovascular diseases. Acta Pharmacol Sin.

[32] Wu J, Dong M, Santos S, Rigatto C, Liu Y, Lin F (2017). Lab-on-a-chip platforms for detection of cardiovascular disease and cancer biomarkers. Sensors.

[33] Aziz Farhanah, Smith Muneera, M Blackburn Jonathan (2019). Autoantibody-Based Diagnostic Biomarkers: Technological Approaches to Discovery and Validation. Autoantibodies and Cytokines.

[34] Bass JJ, Wilkinson DJ, Rankin D, Phillips BE, Szewczyk NJ, Smith K, Atherton PJ (2017). An overview of technical considerations for Western blotting applications to physiological research. Scand J Med Sci Sports.

[35] Welvaert M, Rosseel Y (2013). On the definition of signal-to-noise ratio and contrast-to-noise ratio for fMRI data. PLoS One.

[36] Liu X, Bing T, Shangguan D (2017). Microbead-based platform for multiplex detection of DNA and protein. ACS Appl Mater Interfaces.

[37] Schneider J, Weiss R, Ruhe M, Jung T, Roggenbuck D, Stohwasser R, Schierack P, Rödiger S Open source bioimage informatics tools for the analysis of DNA damage and associated biomarkers. J Lab Precis Med. 2019;4:0. 10.21037/jlpm.2019.04.05.

[38] Liu J, Geng Z, Fan Z, Liu J, Chen H (2019). Point-of-care testing based on smartphone: the current state-of-the-art (2017-2018). Biosens Bioelectron.

[39] Wang MS, Black JC, Knowles MK, Reed SM (2011). C-reactive protein (CRP) aptamer binds to monomeric but not pentameric form of CRP. Anal Bioanal Chem.

