# Supplementary material for: Simultaneous detection and quantification of DNA and protein biomarkers in spectrum of cardiovascular diseases in a microfluidic microbead chip
Source: Anal Bioanal Chem. 2019 Nov 23;411(29):7725–35. doi: 10.1007/s00216-019-02199-x (PMC6881413; doi:10.1007/s00216-019-02199-x)
Supplement: Supplementary file 1 — (PDF 2154 kb) [file 216_2019_2199_MOESM1_ESM.pdf]

Analytical and Bioanalytical Chemistry

Electronic Supplementary Material

Simultaneous detection and quantification of DNA- and protein biomarkers in spectrum of cardiovascular diseases in a microfluidic microbead chip

Franziska Dinter, Michał Burdukiewicz, Peter Schierack, Werner Lehmann, Joerg Nestler, Gregory Dame, Stefan Rödiger

Additional files available under 10.1007/s00216-019-02199-x

Table of content

Supp Sec 1. Workflow of the assay 3

Supp Sec 2. Flow rates of the microfluidic chip 3

Supp Sec 3. Optimization of buffer system...4

Supp Sec 4. Dilution experiments to assess the assay performance 4

Supp Sec 5. Analysis of the fluorescence data 7

Supp Sec 6. Analysis of end point measurements...8

Supp Sec 7. Analysis of dilutions 9

Supp Sec 8. Analysis of kinetics...10

Supp Sec 9. Custom made fit.plot() function to analyze dilution experiments 11

Supp Sec 10. Brief introduction to the digilogger software...13

Supp Sec 11. Supplemental Literature...16

Note

Due to the lack of available data volume, we uploaded the **video** onto a platform.

This can be reached via the following link:

https://www.b-tu.de/owncloud/s/Gowa5GM6G7jKRsw

Supp Sec 1. Workflow of the assay

This supplement section shows a cartoon of the workflow of the assay.

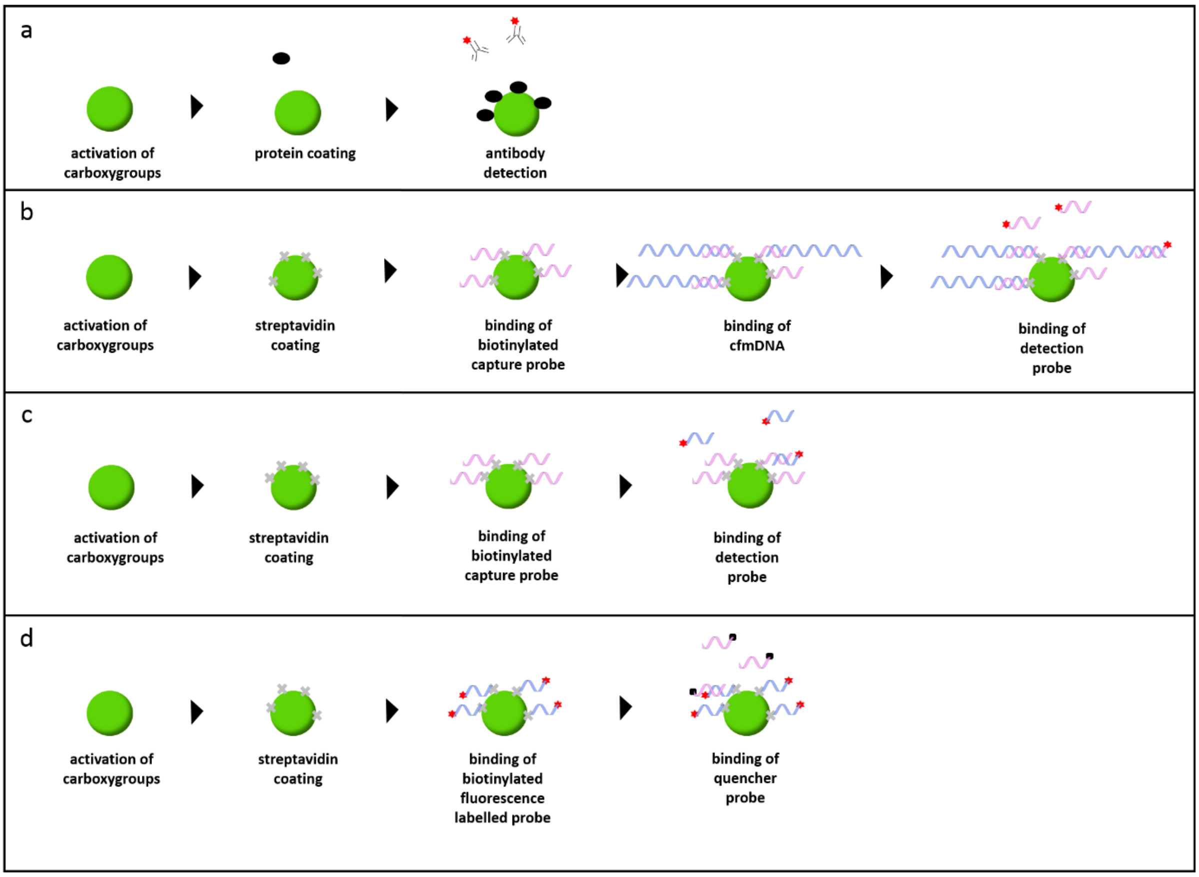

Fig. S1 Cartoon of protein and DNA based assay workflow (a) For protein-based detection of the biomarkers, the microbeads are activated via EDC and coupled to the respective protein in order to be subsequently detected with the corresponding antibody. (b) For the detection of cfmDNA, the microbeads are activated by EDC and coupled with streptavidin. Subsequently, a biotinylated complementary capture probe is bound to the cfmDNA via biotin streptavidin binding. The cfmDNA is bound by the capture probe and in the last step detected by a complementary detection probe, which has a different binding site on the cfmDNA. (c) In order to be able to detect the additional DNA-based biomolecules, the microbeads are activated as in b, coupled with streptavidin and bound to a biotinylated probe. In a further step, this is detected by a fluorescence-labelled complementary probe. (d) In the last assay, a fluorescence-labeled probe is bound to the activated microbeads coupled with streptavidin, whereby the fluorescence is subsequently quenched by a quencher-labeled probe

Supp Sec 2. Flow rates of the microfluidic chip

The Table presents the flow rates of the microfluidic chips during the whole assay procedure.

Table S1 Flow rates of microfluidic chip

pumps	total volume [µL]	application	flow rate [µL/s]	time [sec]
Pump1	20.0	washing buffer	0.1	180
Pump2	20.0	washing buffer	0.1	180
Pump3	40.0	reagent solution	0.1	400
Pump4	40.0	reagent solution	0.1	400
Pump5	20.0	washing buffer	0.1	180
Pump6	20.0	washing buffer	0.1	180

Supp Sec 3. Optimization of buffer system

During assay development the buffer system was optimized for all applied biomarkers.

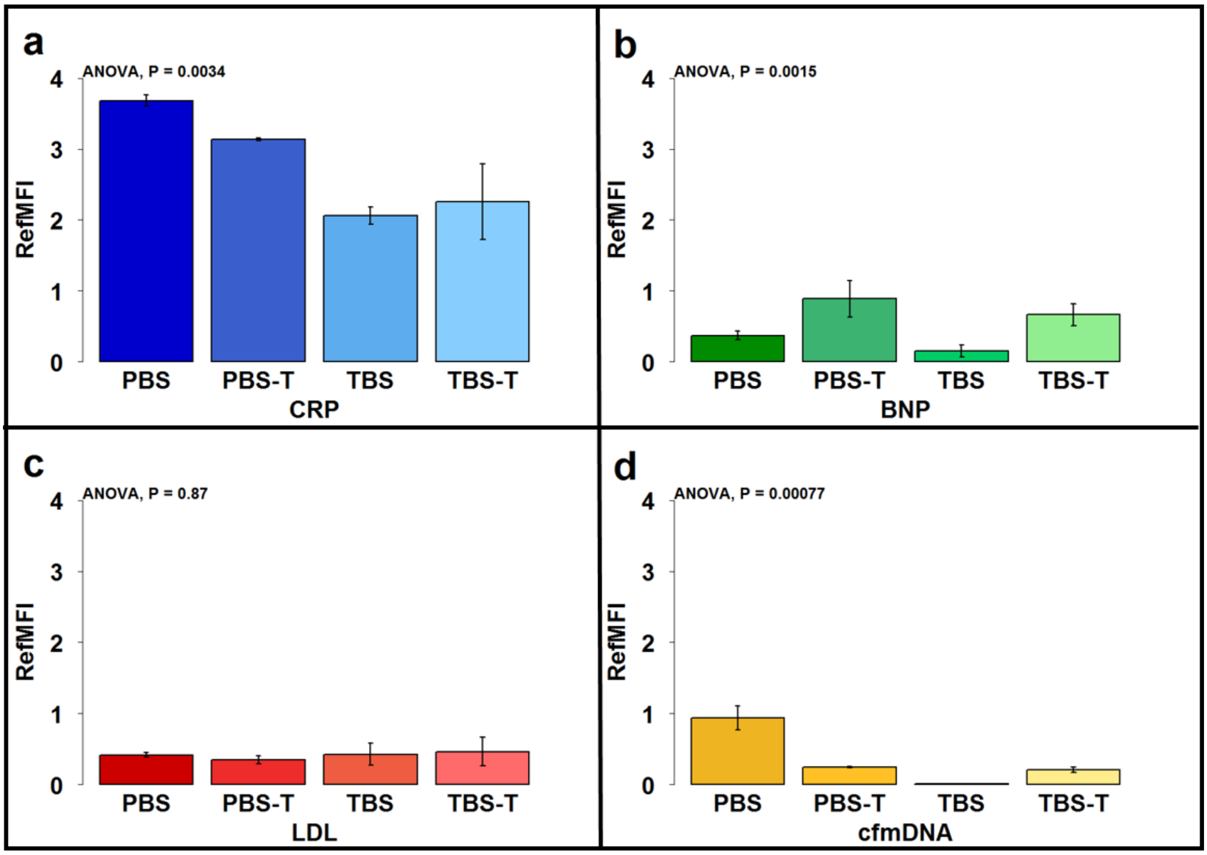

Fig. S2 Selection of the buffer system for the simultaneous detection of protein and DNA based biomarkers. Four different buffer systems were tested for the development of the proof of concept. To find out which of these buffers is suitable for simultaneous detection of protein and DNA based biomarkers, all biomarkers were detected individually in all buffer systems CRP (a), BNP (b), LDL (c), cfmDNA (d)

Supp Sec 4. Dilution experiments to assess the assay performance

The following figures show the different dilution series of all biomarkers in the chip system and in the multiwell plate. The first figure S3 illustrates the dilution series of cfmDNA under optimal conditions. The figure below S4 displays the dilution series under buffer conditions for CRP, BNP and LDL. The last figure S5 presents the dilution series of the biomarkers in spiked human serum.

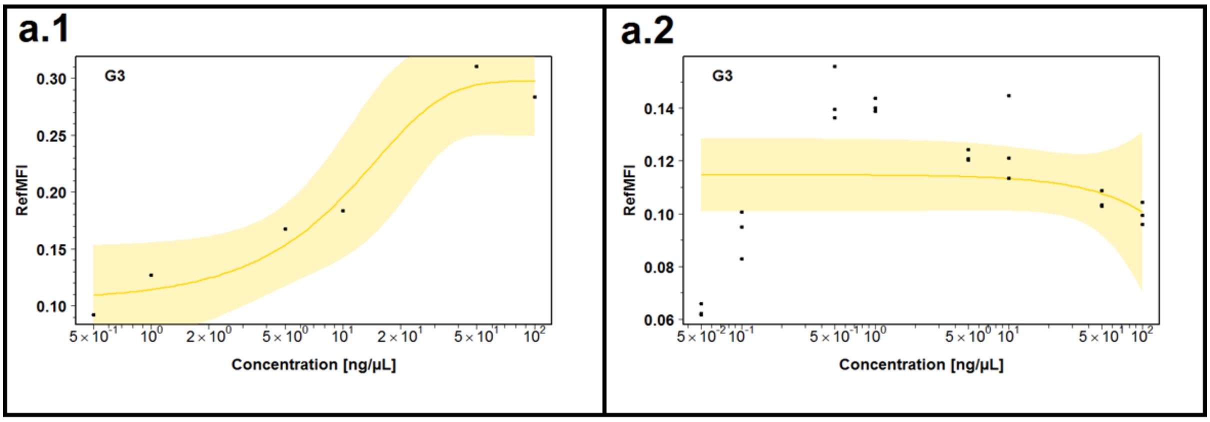

Fig. S3 Serial dilution of cfmDNA in chip and multiwell plate; cfmDNA was detected using different concentrations from 0.5 ng/μL to 100 ng/μL by a detection probe in the chip (a.1) and in a multiwell plate (a.2)

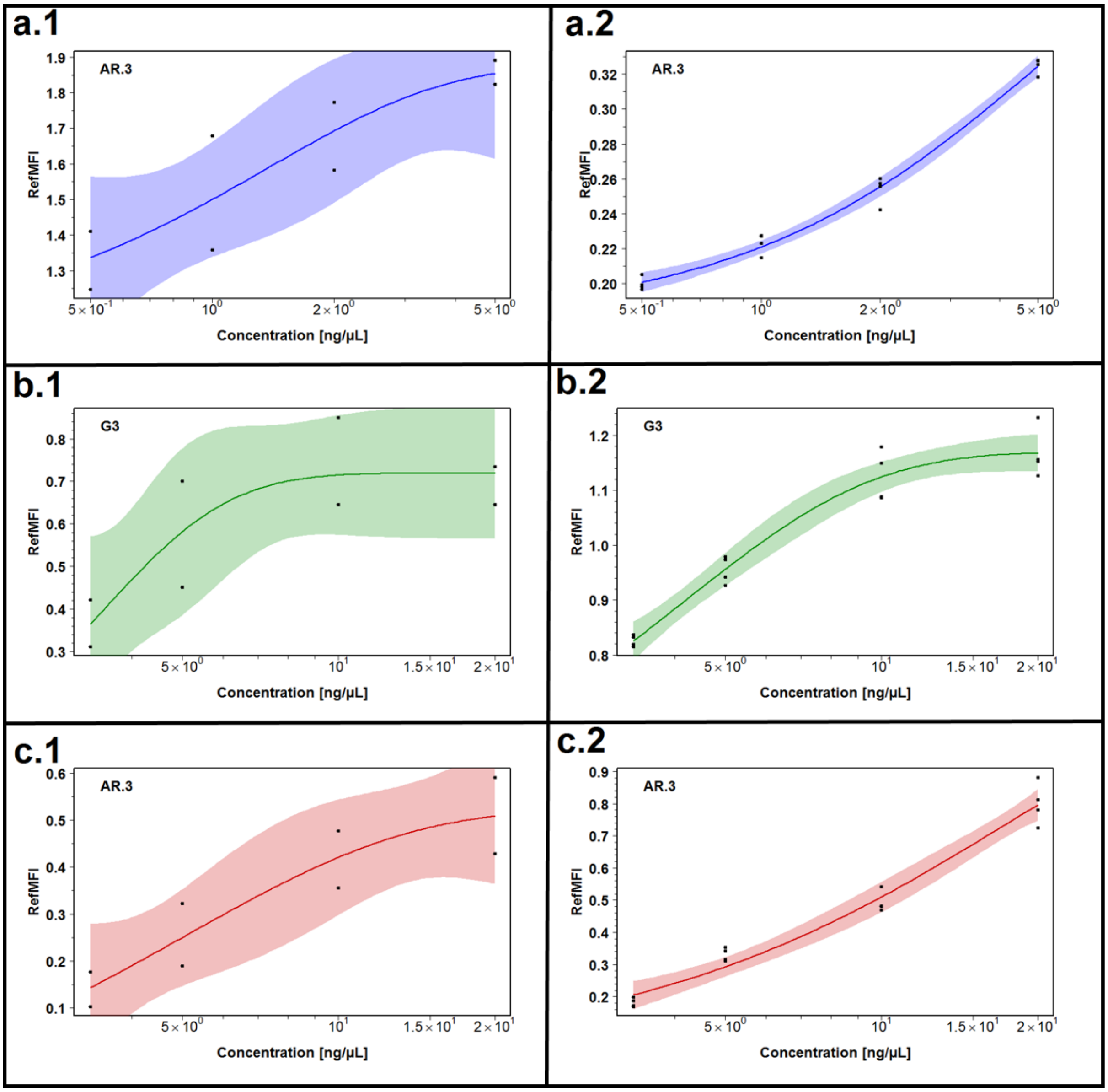

Fig. S4 Simultaneous detection by dilution series of the biomarkers anti - CRP, anti - BNP and anti – LDL. The biomarkers CRP (a), BNP (b) and LDL (c) were detected simultaneously by dilution series of 0.5 ng/μL to 5 ng/μL (CRP) and 3.33 ng/μL to 20 ng/μL (BNP and LDL) in the chip (a-c.1) and in the multiwell plate (a-c.2)

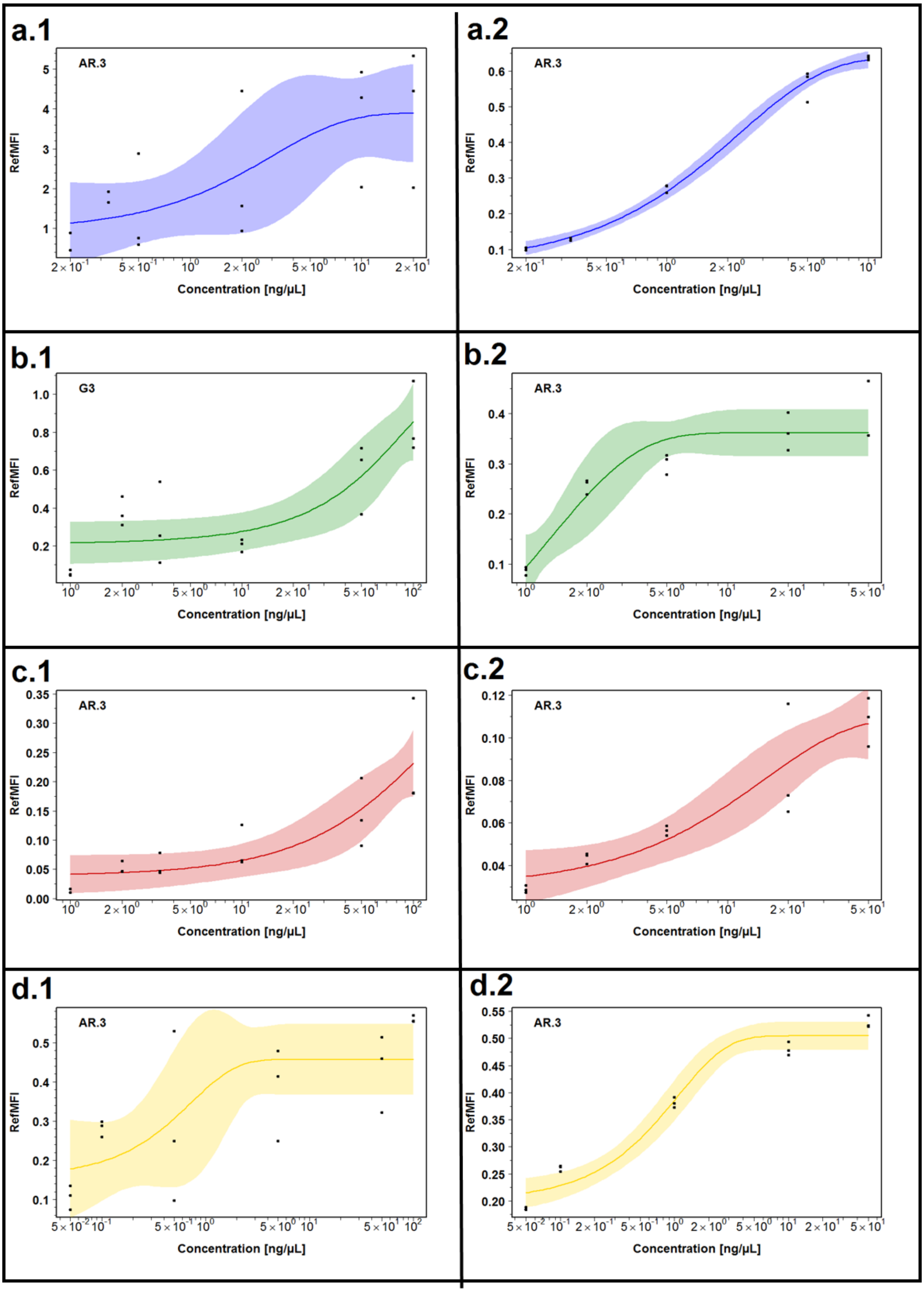

Fig. S5 Simultaneous detection by dilution series of the biomarkers anti - CRP, anti - BNP anti - LDL and cfmDNA in spiked human serum. The biomarkers anti - CRP (a), anti - BNP (b) and anti - LDL (c) were detected simultaneously by dilution series of 0.2 ng/µL to 10 ng/µL (CRP) and 1 ng/µL to 50 ng/µL (BNP and LDL) in the chip (a-c.1) and in the multiwell plate (a-c.2). cfmDNA could be detected over a range from 0.05 ng/µL to 50 ng/µL (d.1/d.2)

Supp Sec 5. Analysis of the fluorescence data

All numerical raw data, derived by feature extraction using bioimage informatics algorithms implemented in the FastFluoScan software [26]. Since we used fluorescence for quantification, it is critical to work in the linear range of fluorescence intensity. In previous studies [40] we used the VideoScan platform to study the surface functionalization of poly(methylmethacrylate) microbeads. During this study we used the FastFluoScan software (see [26] section 2.1 - 2.2) from the VideoScan platform. There were showed that it is possible to use the platform at least between 0 - 15 to work in linear the range of fluorescence intensity. This is possible for the following reasons.

- Measurement of ligands on microbeads: First the image is taken in the ligand channel with initial exposure time (e.g. 1000 ms) and first determination of the ligand values (refMFI) at the microbeads. In further steps, the measurement signals are referenced to the coding dyes (green and blue channel). For details see [26] section 2.1 - 2.2.
- If a pixel in the ligand measuring range of a microbead is overexposed (saturated), then an image with half the exposure time is taken in the ligand channel. This image is then used to determine the ligand values for the previously overexposed microbeads (adaptive algorithm).
- Due to the linearity of the CCD sensors, the exposure time is used linearly to determine the ligand value. Put simply: with the same grey value in the image, halving the exposure time means doubling the ligand value. A halving of the exposure time produces a halving of the gray values in the captured image; previously overexposed areas in the image can now provide an evaluable gray value.
- This halving could be continued as often as desired as long as ligands of microbeads are overexposed. This leads to a corresponding increase in the ligand values. The number of halving is configurable and currently limited to a maximum of 8 iteration steps.

All numerical raw data were analyzed with the computing language R [28].

Supp Sec 6. Analysis of end point measurements

The following workflow was used for the analysis of the raw data.

Data processing steps	*Example*
1. Capture image data with the reader platform	Measure images with VideoScan
2. Process image data and extract features as numerical values	Use FastFluoScan to calculate the features

3. Import the numerical values for data processing

Projektname	Projekt009039-14-06-2018_FranziD_Chip64_alle mix_CRP						
Profil name	FDinter_4x_C0424-C0435_FlexFlowChip_2erEinlegerahmen						
LOT							
blockweise Ausgabe:							
1. Block	Ergebnisse						
2. Block	Mittelwerte						
3. Block	Ereignisanzahlen						
4. Block	Standardabweichungen						
5. Block	CV-Werte						
6. Block	Well-Informationen und Ergebnisnamen						
Ergebnisse							
ZeilenIndex	SpaltenIndex	Well	ID	Name	Ergebnis 1	Ergebnis 2	Ergebnis 3
0	0	Slide1_A1			0,09482679	0,12674074	0,08599521
1	0	Slide2_A1					
Mittelwerte							
ZeilenIndex	SpaltenIndex	Well	ID	Name	Mittelwert 1	Mittelwert 2	Mittelwert 3
0	0	Slide1_A1			0,09482679	0,12674074	0,08599521
1	0	Slide2_A1					

4. Manual reshaping of data

CRP							
	Chip Nummer	LOT Nr.	Datum	Konzentration	MP-Population	Puffer	CRP
	C64	021-70461	14.06.2018	1:50	CO430	PBS	6,01084479
	C66	021-70461	14.06.2018	1:50	CO430	PBS	3,65975277
	C68	021-70461	19.09.2018	1:50	CO430	PBS	3,77169388
	C99	021-70661	25.07.2018	1:50	CO430	PBS	3,61582448
Mittelwert							3,68242371
	C151	021-80491	01.11.2018	1:50	CO430	PBS-T	3,12265428
	C152	021-80491	01.11.2018	1:50	CO430	PBS-T	3,15197628
Mittelwert							3,13731528
	C139	021-80411	24.10.2018	1:50	CO430	TBS	1,97400126
	C140	021-80411	24.10.2018	1:50	CO430	TBS	2,14522643
Mittelwert							2,059613845
	C119	021-70661	19.09.2018	1:50	CO430	TBS-T	2,86781855
	C192	021-80521	09.01.2018	1:50	CO430	TBS-T	5,16865614
	C237	021-80521	30.01.2019	1:50	CO430	TBS-T	1,85114618
	C243	021-80521	01.02.2019	1:50	CO430	TBS-T	2,05310672
Mittelwert							2,25735715

5. Analysis of endpoint measurements using the barplot() function

```
# R code for the analysis
anova()
barplot(my.data)
```

R Console | tbl_df 4 x 5

CRP <dbl>	BNP <dbl>	LDL <dbl>	cfmDNA <dbl>	SAv <dbl>
2.867819	0.7593168	NA	0.03562623	0.13002442
5.168656	0.3418535	NA	0.12055005	0.07802065
1.851146	0.2424198	0.02072357	0.02197410	0.02097622
2.053107	0.1677690	0.01569421	0.01894027	0.01211734

4 rows

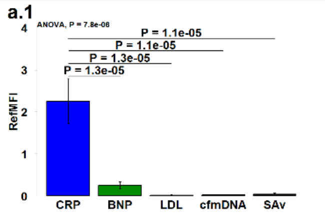

6. Analysis and visualization of numeric data with digilogger

Supp Sec 7. Analysis of dilutions

This workflow was used to analyze dilution experiments. For this purpose the fit.plot() function (Supp Sec 8) was used.

Data processing steps	*Example*
1. Capture image data with the reader platform	Measure images with VideoScan
2. Process image data and extract features as numerical values	Use FastFluoScan to calculate the features
3. Import the numerical values for data processing	(see spreadsheet below)
4. Maunal reshaping of data	(see table below)
5. Analysis of dilutions using the fit.plot() function	# R code for the analysis<br>anova()<br>fit.plot(my.data)
6. Analysis and visualization of numeric data with digilogger	

Step 3 example:

Projektname	Projekt009383-19-09-2018_FranziD_Chip119_CRP_50_nl						
Profil name	FDinter_4x_C0424-C0435_FlexFlowChip_2erEinlegerahmen						
LOT							
blockweise Ausgabe:							
1. Block	Ergebnisse						
2. Block	Mittelwerte						
3. Block	Ereignisanzahlen						
4. Block	Standardabweichungen						
5. Block	CV-Werte						
6. Block	Well-Informationen und Ergebnisnamen						
Ergebnisse							
ZeilenIndex	SpaltenIndex	Well	ID	Name	Ergebnis 1	Ergebnis 2	Ergebnis 3
0	0	Slide1_A1			unzureichend	0,03562623	0,03379137
1	0	Slide2_A1					
Mittelwerte							
ZeilenIndex	SpaltenIndex	Well	ID	Name	Mittelwert 1	Mittelwert 2	Mittelwert 3
0	0	Slide1_A1				0,03562623	0,03379137
1	0	Slide2_A1					

Step 4 example:

Verdünnung	rep1	rep2	rep2
20,000	2,86781855	1,85114618	2,05310672
10,000	2,78325459	1,67977154	1,83697208
5,000	1,18775406	1,59429272	
2,000	2,05219539	1,16063652	1,41141852
1,000	1,52126517	1,02893542	1,04054969
0,500	0,55400083	0,75090714	
0,400	0,31142522	0,32588227	
0,333	0,56411479	0,42196009	
0,200	0,17679874	0,09729003	

Step 5 output:

```
[1] 5 4
Classes 'tbl_df', 'tbl' and 'data.frame':	5 obs. of  4 variables:
 $ Verdünnung: num  10 5 1 0.333 0.2
 $ rep1      : num  0.643 0.584 0.279 0.133 0.105
 $ rep2      : num  0.638 0.593 0.259 0.125 0.098
 $ rep3      : num  0.631 0.512 0.278 0.132 0.105
   Verdünnung          rep1             rep2             rep3       
 Min.   : 0.200   Min.   :0.1050   Min.   :0.0980   Min.   :0.1047  
 1st Qu.: 0.333   1st Qu.:0.1328   1st Qu.:0.1253   1st Qu.:0.1320  
 Median : 1.000   Median :0.2785   Median :0.2595   Median :0.2780  
 Mean   : 3.307   Mean   :0.3486   Mean   :0.3428   Mean   :0.3317  
 3rd Qu.: 5.000   3rd Qu.:0.5842   3rd Qu.:0.5928   3rd Qu.:0.5124  
 Max.   :10.000   Max.   :0.6426   Max.   :0.6384   Max.   :0.6313  
null device 
          1 
```

a.1

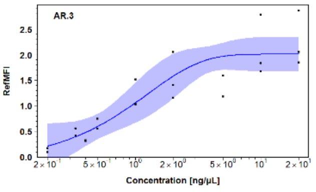

Supp Sec 8. Analysis of kinetics

Data processing steps	*Example*
1. Capture image data with the reader platform	Measure images with VideoScan
2. Process image data and extract features as numerical values	Use FastFluoScan to calculate the features
3. Import the numerical values for data processing	(see table below)

Kinetik

Messwerte

Zeile A

Zeit [s]	Zyklus	Wert von C04	Wert von C04	Wert von C04	Wert von C04
0	0		0,0861866	0,0924682	0,08789144
159	1		0,08524419	0,08691446	0,08773181
303	2		0,08577691	0,08750258	0,0882059
453	3		0,08599033	0,08755118	0,08655167
594	4		0,08587341	0,08647341	0,08799657
741	5		0,08410897	0,08618051	0,08661796
882	6		0,0845017	0,08278085	0,08354392
1036	7		0,08433451	0,08368913	0,08427967
1179	8		0,08482004	0,085344	0,08567095
1329	9		0,0809258	0,08169222	0,08140601
1471	10		0,08111927	0,07983285	0,07887271
1619	11		0,08143004	0,08266234	0,08141756
1761	12		0,08146886	0,079176	0,08141711
1912	13		0,08186229	0,07776689	0,07859606
2056	14		0,08154679	0,08078436	0,07876528
2205	15		0,08251368	0,08199667	0,08198373
2352	16		0,08247409	0,08033393	0,07786309
2499	17		0,08221432	0,07856073	0,07931526
2646	18		0,08169172	0,08107325	0,07935049
2786	19		0,08100694	0,07720332	0,07855089
2936	20		0,081283	0,07832479	0,07965441
3077	21		0,08109232	0,07817128	0,0804422
3224	22		0,07930012	0,07790967	0,07974703
3366	23		0,08071389	0,07818116	0,08028489

4. Reshaping of data

CRP 1:1000

	Zeit	Chip	StreptavidinCl
	0	0	0
	128	0	0
	259	0	0
	400	0,67233257	0,00590916
	572	0,7656603	0,00788861
	705	0,85744638	0,01029934
	846	0,9332198	0,01051929
	979	1,00275195	0,00899059
	1120	1,06457839	0,01145154
	1262	1,11922688	0,01150198
	1393	1,16789647	0,01032795
	1540	1,21687321	0,00855093
	1684	1,26571907	0,01045645
	1829	1,30949041	0,00883822
	1981	1,33852927	0,01053872
	2128	1,38010051	0,00836163
	2271	1,41026634	0,00904529
	2406	1,43482225	0,01066668
	2540	1,46230712	0,01140619
	2671	1,49018504	0,01168938
	2802	1,51079201	0,01088934
	2937	1,53235228	0,01197441
	3082	1,544	0,00886183
	3216	1,55591724	0,007853
	3372	1,57500101	0,01124746

5. Analysis of dilutions using the plot() function

```
# R code for the analysis
anova()
plot(my.data)
lines()
```

```
[1] 25  5
Classes 'tbl_df', 'tbl' and 'data.frame':       25 obs. of  5 variables:
 $ Zeit              : num  0 128 259 400 572 ...
 $ Chip              : num  0 0 0 0.672 0.766 ...
 $ StreptavidinChip  : num  0 0 0 0.00591 0.00789 ...
 $ Platte            : num  0.146 0.181 0.204 0.222 0.237 ...
 $ StreptavidinPlatte: num  0.078 0.0786 0.0796 0.0773 0.0774 ...
      Zeit           Chip          StreptavidinChip        Platte         StreptavidinPlatte
 Min.   :   0   Min.   :0.0000   Min.   :0.000000   Min.   :0.1465   Min.   :0.07216
 1st Qu.: 846   1st Qu.:0.9332   1st Qu.:0.008362   1st Qu.:0.2598   1st Qu.:0.07478
 Median :1684   Median :1.2657   Median :0.010299   Median :0.3084   Median :0.07566
 Mean   :1685   Mean   :1.1044   Mean   :0.008691   Mean   :0.2917   Mean   :0.07601
 3rd Qu.:2540   3rd Qu.:1.4623   3rd Qu.:0.010889   3rd Qu.:0.3342   3rd Qu.:0.07730
 Max.   :3372   Max.   :1.5750   Max.   :0.011974   Max.   :0.3546   Max.   :0.07957
null device
          1
```

a

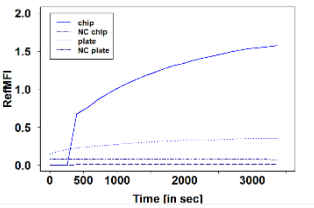

Supp Sec 9. Custom made fit.plot() function to analyze dilution experiments

The following code of the custom made fit.plot() function was used to analyze the dilution experiments.

```
# R function for automatic curve fitting and plotting

fit.plot <- function(df, color = "red", points = TRUE, add = FALSE) {

# reshape2::melt was was used to reshape to data from wide format to

# long format. drc::drm was used to fit the non-linear model to the data
  df <- reshape2::melt(df, id.vars = "Verdünnung")[, c(1,3)]

  fm.A <- drc::drm (value ~ Verdünnung, data = df, fct = G.3())
  fm.B <- drc::drm(value ~ Verdünnung, data = df, fct = G.4())
  fm.C <- drc::drm(value ~ Verdünnung, data = df, fct = AR.3())
# Selection of the best model done based on the Schwarz's Bayesian

# criterion information criterion (BIC). The model with the lowest BIC was

# used for further analysis.
  BIC.fm.A <- BIC(fm.A)
  BIC.fm.B <- BIC(fm.B)
  BIC.fm.C <- BIC(fm.C)

  models <- c(BIC.fm.A, BIC.fm.B, BIC.fm.C)

  minumum <- min(models)

  opt_model <- which(models == minumum)
# The optimal model was plotted along the 95% confidence intervals

# for model parameters.


  if (opt_model == 1) {
    plot(fm.A, add = add, col = color, xlab = "",
         ylab = "", axes =FALSE, type = c("confidence"),
         font = 2, font.axis = 2, font.lab = 2,
         cex.lab= 2, cex.axis = 3, cex=3)
  }
  if (opt_model == 2) {
    plot(fm.B, add = add, col = color, xlab = "",
         ylab = "", axes =FALSE, type = c("confidence"),
         font = 2, font.axis = 2, font.lab = 2,
         cex.lab= 2, cex.axis = 3, cex=3)
  }
```

```
  if (opt_model == 3) {
    plot(fm.C, add = add, col = color, xlab = "",
         ylab = "", axes =FALSE, type = c("confidence"),
         font = 2, font.axis = 2, font.lab = 2,
         cex.lab= 2, cex.axis = 3, cex=3)
  }

  if(points) points(df$Verdünnung, df$value,
                    pch = 19, cex = 1)

  model_names <- c("G3", "G.4", "AR.3")

  legend("topleft", legend = model_names[opt_model],
         bty = "n", cex = 2)
}
```

Supp Sec 10. Brief introduction to the digilogger software

Cardiovascular diseases are one of the main reasons for adult mortality in western world, leading to huge liability on healthcare system. Lifestyle, genetic disposition, deficiency of clearly defined risk assessment criteria, as well as high occurrences of misdiagnosis directly contribute to this issue. Due to this fact a rapid and simultaneously detection of biomarkers is necessary. We started to integrate biomarker detection in a microfluidic microbead chip system that is read-out with our VideoScan technology as proposed earlier [26]. In addition, we developed software, which can be used for the analysis. We integrated the biomarkers in different detection methods (hybridization assays and microbead ELISA) and tested those methods as multiplex assays.

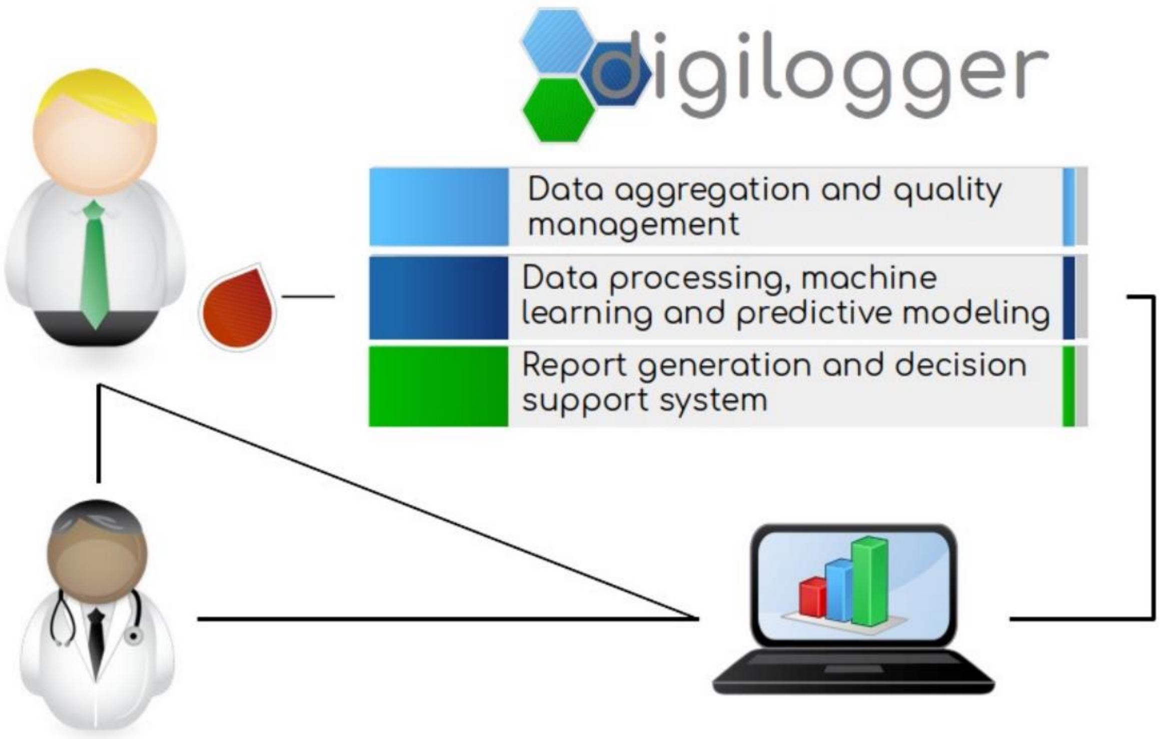

The digilogger app automatically reads and processes measured values from POCT devices. The digilogger software is a graphical front end for the analysis of multiparametric measurement data. The Shiny technology was developed for the development of stand-alone applications. The software is relatively easy to use. No knowledge of HTML, CSS and JavaScript is required. Shiny does not require any external web server software to be installed on the server. Apart from R and Shiny, only other basic R packages are required.

Installation:

digilogger is available as the latest development version of the code by using the devtools R package.

Requirements: The digilogger software requires a functional R environment (version 3.3 or later) and a working installation of the shiny package. Our digilogger standalone graphical user interface allows the analysis of POCT data without installing the package on a server. Since the digilogger

software is based on modern web technologies like HTML5 it will run on any modern web browser. Additionally, the software works when run in RStudio or RKWard (version 0.7.0z+0.7.1+devel1). The digilogger software works platform independent (tested on Linux and Windows).

```
# Start R
# Install devtools, if you haven't already.
install.packages("devtools")

devtools::install_github("michbur/digilogger")
```

Using the software:

```
# Start R
digilogger::digilogger_gui()
```

An important implementation for this software is the possibility to visualize the measurement data interactively and to get an insight how the measurement data develops over a period of time.

The raw data are not changed but are entered into a database uniquely identifiable with IDs and hashes.

Currently, only measurements from the VideoScan platform [26] are supported. However, users can create their own import functions by adapting the functions in the vs.import.R file of the digilogger package.

After processing, the raw data are qualitatively checked and presented with interactive diagrams and tables.

During the analysis using POCTs, data is generated continuously. This is particularly the case with multiparametric systems. With multiparametric systems such as VideoScan technology, the following values, among others, are recorded simultaneously in one sample:

- DNA and protein biomarkers,
- Time of measurement,
- Age
- Gender.

In the **Files** tab the input files can be checked for their plausibility. For each file the file name, its hash value (md5sum), the profile name and the ID are known. This information can be used to check whether an incorrect assignment has been satisfied.

The **Raw Data** tab contains an interactive table for quick viewing of input data. The data can be filtered and sorted. These steps have no influence on the further analysis steps.

In principle, the DNA and protein biomarkers can already be validated and clinically proven or are still at an experimental stage. The latter are of particular interest because they can be assumed to be suitable for diagnostic applications in the future.

On the basis of the measured values, classes (e.g., "normal", "borderline", "diseased") can in principle be determined. This knowledge can be used for further purposes.

The aim of the digilogger technology is the continuous acquisition of measurement data and the collection of measurement data in a database which is used for machine learning. Models are continuously developed and compared to previous knowledge. For example, threshold values for new (experimental) biomarkers are to be determined automatically. In addition, the software will support the classification of patients.

Supp Sec 11. Supplemental Literature

1. Upadhyay RK (2015) Emerging Risk Biomarkers in Cardiovascular Diseases and Disorders. J Lipids 2015:1–50

2. National Institutes of HealthDirector's Initiative on Biomarkers and Surrogate Endpoints (2001) Biomarkers and surrogate endpoints: Preferred definitions and conceptual framework. Clin Pharmacol Ther 69:89–95

3. McDonnell B, Hearty S, Leonard P, O'Kennedy R (2009) Cardiac biomarkers and the case for point-of-care testing. Clin Biochem 42:549–561

4. Dhingra R, Vasan RS (2017) Biomarkers in cardiovascular disease: Statistical assessment and section on key novel heart failure biomarkers. Trends Cardiovasc Med 27:123–133

5. E. Berezin A (2015) Circulating Cell-Free Mitochondrial DNA as Biomarker of Cardiovascular risk: New Challenges of Old Findings. Angiol Open Access. doi: 10.4172/2329-9495.1000161

6. Albert MA (2011) Biomarkers and Heart Disease. J Clin Sleep Med. doi: 10.5664/jcsm.1342

7. Peng S, Hong T, Liang W, Liu W, Chen C (2019) A multichannel microchip containing 16 chambers packed with antibody-functionalized beads for immunofluorescence assay. Anal Bioanal Chem 411:1579–1589

8. Nakayama H, Otsu K (2018) Mitochondrial DNA as an inflammatory mediator in cardiovascular diseases. Biochem J 475:839–852

9. Berezin AE (2016) DTOhI: 1e0.21C76e7/l2l1-72F-0r4e79e.100M068itochondrial DNA: A Novel Biomarker of Cardiovascular Risk? Transl Biomed 4

10. Amann RI, Devereux R, Stahl' DA (1990) Combination of 16S rRNA-Targeted Oligonucleotide Probes with Flow Cytometry for Analyzing Mixed Microbial Populations. APPL Env MICROBIOL 56:7

11. Meier LA, Binstadt BA (2018) The Contribution of Autoantibodies to Inflammatory Cardiovascular Pathology. Front Immunol. doi: 10.3389/fimmu.2018.00911

12. Liang KP, Maradit-Kremers H, Crowson CS, Snyder MR, Therneau TM, Roger VL, Gabriel SE (2009) Autoantibodies and the Risk of Cardiovascular Events. J Rheumatol 36:2462–2469

13. Müller J, Wallukat G, Schimke I (2017) Autoantibody-Directed Therapy in Cardiovascular Diseases. In: Heart Rheum. Autoimmune Inflamm. Dis. Elsevier, pp 659–679

14. Frostegård J (2002) Autoimmunity, oxidized LDL and cardiovascular disease. Autoimmun Rev 1:233–237

15. O'Neill SG, Isenberg DA, Rahman A (2007) Could antibodies to C-reactive protein link inflammation and cardiovascular disease in patients with systemic lupus erythematosus? Ann Rheum Dis 66:989–991

16. Wetterö J, Nilsson L, Jonasson L, Sjöwall C (2009) Reduced serum levels of autoantibodies against monomeric C-reactive protein (CRP) in patients with acute coronary syndrome. Clin Chim Acta 400:128–131

17. O'Neill SG, Giles I, Lambrianides A, Manson J, D'Cruz D, Schrieber L, March LM, Latchman DS, Isenberg DA, Rahman A (2010) Antibodies to apolipoprotein A-I, high-density lipoprotein, and C-reactive protein are associated with disease activity in patients with systemic lupus erythematosus. Arthritis Rheum 62:845–854

18. Wilson PWF, Ben-Yehuda O, McNamara J, Massaro J, Witztum J, Reaven PD (2006) Autoantibodies to oxidized LDL and cardiovascular risk: The Framingham Offspring Study. Atherosclerosis 189:364–368

19. Vaarala O (2000) Autoantibodies to modified LDLs and other phospholipid-protein complexes as markers of cardiovascular diseases. J Intern Med 247:381–384

20. Iseme RA, McEvoy M, Kelly B, Agnew L, Walker FR, Handley T, Oldmeadow C, Attia J, Boyle M (2017) A role for autoantibodies in atherogenesis. Cardiovasc Res 113:1102–1112

21. Rödiger S, Liebsch C, Schmidt C, Lehmann W, Resch-Genger U, Schedler U, Schierack P (2014) Nucleic acid detection based on the use of microbeads: a review. Microchim Acta 181:1151–1168

22. Streit P, Nestler J, Shaporin A, Graunitz J, Otto T (2018) Design methodology and results evaluation of a heating functionality in modular lab-on-chip systems. J Micromechanics Microengineering 28:064001

23. Schumacher S, Nestler J, Otto T, et al (2012) Highly-integrated lab-on-chip system for point-of-care multiparameter analysis. Lab Chip 12:464–473

24. Geidel S, Peransi Llopis S, Rodrigo M, de Diego-Castilla G, Sousa A, Nestler J, Otto T, Gessner T, Parro V (2016) Integration of an Optical Ring Resonator Biosensor into a Self-Contained Microfluidic Cartridge with Active, Single-Shot Micropumps. Micromachines 7:153

25. Rödiger S, Ruhland M, Schmidt C, Schröder C, Grossmann K, Böhm A, Nitschke J, Berger I, Schimke I, Schierack P (2011) Fluorescence Dye Adsorption Assay to Quantify Carboxyl Groups on the Surface of Poly(methyl methacrylate) Microbeads. Anal Chem 83:3379–3385

26. Rödiger S, Schierack P, Böhm A, et al (2012) A Highly Versatile Microscope Imaging Technology Platform for the Multiplex Real-Time Detection of Biomolecules and Autoimmune Antibodies. In: Seitz H, Schumacher S (eds) Mol. Diagn. Springer Berlin Heidelberg, Berlin, Heidelberg, pp 35–74

27. Gallizia A, de Lalla C, Nardone E, Santambrogio P, Brandazza A, Sidoli A, Arosio P (1998) Production of a Soluble and Functional Recombinant Streptavidin inEscherichia coli. Protein Expr Purif 14:192–196

28. R Core Team (2018) R: A Language and Environment for Statistical Computing. R Found Stat Comput https://www.R-project.org/

29. Ritz C, Baty F, Streibig JC, Gerhard D (2015) Dose-Response Analysis Using R. PLOS ONE 10:e0146021

30. Prosperi M, Min JS, Bian J, Modave F (2018) Big data hurdles in precision medicine and precision public health. BMC Med Inform Decis Mak. doi: 10.1186/s12911-018-0719-2

31. Adamcova M, Šimko F (2018) Multiplex biomarker approach to cardiovascular diseases. Acta Pharmacol Sin 39:1068–1072

32. Wu J, Dong M, Santos S, Rigatto C, Liu Y, Lin F (2017) Lab-on-a-Chip Platforms for Detection of Cardiovascular Disease and Cancer Biomarkers. Sensors 17:2934

33. Aziz F, Smith M, M Blackburn J (2019) Autoantibody-Based Diagnostic Biomarkers: Technological Approaches to Discovery and Validation. Autoantibodies Cytokines. doi: 10.5772/intechopen.75200

34. Bass JJ, Wilkinson DJ, Rankin D, Phillips BE, Szewczyk NJ, Smith K, Atherton PJ (2017) An overview of technical considerations for Western blotting applications to physiological research. Scand J Med Sci Sports 27:4–25

35. Welvaert M, Rosseel Y (2013) On the Definition of Signal-To-Noise Ratio and Contrast-To-Noise Ratio for fMRI Data. PLoS ONE 8:e77089

36. Liu X, Bing T, Shangguan D (2017) Microbead-Based Platform for Multiplex Detection of DNA and Protein. ACS Appl Mater Interfaces 9:9462–9469

37. Schneider J, Weiss R, Ruhe M, Jung T, Roggenbuck D, Stohwasser R, Schierack P, Rödiger S (2019) Open source bioimage informatics tools for the analysis of DNA damage and associated biomarkers. J Lab Precis Med 21–21

38. Liu J, Geng Z, Fan Z, Liu J, Chen H (2019) Point-of-care testing based on smartphone: The current state-of-the-art (2017-2018). Biosens Bioelectron 132:17–37

39. Wang MS, Black JC, Knowles MK, Reed SM (2011) C-reactive protein (CRP) aptamer binds to monomeric but not pentameric form of CRP. Anal Bioanal Chem 401:1309–1318

40. Rödiger S, Ruhland M, Schmidt C, Schröder C, Grossmann K, Böhm A, Nitschke J, Berger I, Schimke I, Schierack P (2011) Fluorescence Dye Adsorption Assay to Quantify Carboxyl Groups on the Surface of Poly(methyl methacrylate) Microbeads. Anal Chem 83:3379–3385 . doi: 10.1021/ac103277s
